# Targeted inhibition of Wnt signaling with a *Clostridioides difficile* toxin B fragment suppresses breast cancer tumor growth

**DOI:** 10.1371/journal.pbio.3002353

**Published:** 2023-11-09

**Authors:** Aina He, Songhai Tian, Oded Kopper, Daniel J. Horan, Peng Chen, Roderick T. Bronson, Ren Sheng, Hao Wu, Lufei Sui, Kun Zhou, Liang Tao, Quan Wu, Yujing Huang, Zan Shen, Sen Han, Xueqing Chen, Hong Chen, Xi He, Alexander G. Robling, Rongsheng Jin, Hans Clevers, Dongxi Xiang, Zhe Li, Min Dong

**Affiliations:** 1 Department of Oncology, Shanghai Jiaotong University Affiliated Sixth People’s Hospital, Shanghai, People’s Republic of China; 2 Department of Urology, Boston Children’s Hospital, Harvard Medical School, Boston, Massachusetts, United States of America; 3 Department of Microbiology and Department of Surgery, Harvard Medical School, Boston, Massachusetts, United States of America; 4 State Key Laboratory of Natural and Biomimetic Drugs, Department of Molecular and Cellular Pharmacology, School of Pharmaceutical Sciences, Peking University, Beijing, People’s Republic of China; 5 Hubrecht Institute, Royal Netherlands Academy of Arts and Sciences and University Medical Center Utrecht, Utrecht, the Netherlands; 6 Department of Anatomy & Cell Biology, Indiana University School of Medicine, Barnhill, Indianapolis, United States of America; 7 Department of Physiology and Biophysics, University of California, Irvine, California, United States of America; 8 Rodent Histopathology, Harvard Medical School, Boston, Massachusetts, United States of America; 9 Kirby Neurobiology Center, Boston Children’s Hospital, Department of Neurology, Harvard Medical School, Boston, Massachusetts, United States of America; 10 Department of Vascular Biology, Boston Children’s Hospital, Boston, Massachusetts, United States of America; 11 Central Laboratory of Medical Research Centre, The First Affiliated Hospital of USTC, Division of Life Sciences and Medicine, University of Science and Technology of China, Hefei, Anhui, People’s Republic of China; 12 Division of Genetics, Department of Medicine, Brigham and Women’s Hospital, Boston, Massachusetts, United States of America; 13 Department of Medicine, Harvard Medical School, Boston, Massachusetts, United States of America; 14 State Key Laboratory of Oncogenes and Related Genes, Shanghai Cancer Institute, Renji Hospital, Shanghai Jiao Tong University School of Medicine, Shanghai, People’s Republic of China; 15 Department of Biliary-Pancreatic Surgery, Renji Hospital, Shanghai Jiao Tong University School of Medicine, Shanghai, People’s Republic of China; B.C. Cancer Agency, CANADA

## Abstract

Wnt signaling pathways are transmitted via 10 homologous frizzled receptors (FZD1-10) in humans. Reagents broadly inhibiting Wnt signaling pathways reduce growth and metastasis of many tumors, but their therapeutic development has been hampered by the side effect. Inhibitors targeting specific Wnt-FZD pair(s) enriched in cancer cells may reduce side effect, but the therapeutic effect of narrow-spectrum Wnt-FZD inhibitors remains to be established in vivo. Here, we developed a fragment of *C*. *difficile* toxin B (TcdB^FBD^), which recognizes and inhibits a subclass of FZDs, FZD1/2/7, and examined whether targeting this FZD subgroup may offer therapeutic benefits for treating breast cancer models in mice. Utilizing 2 basal-like and 1 luminal-like breast cancer models, we found that TcdB^FBD^ reduces tumor-initiating cells and attenuates growth of basal-like mammary tumor organoids and xenografted tumors, without damaging Wnt-sensitive tissues such as bones in vivo. Furthermore, FZD1/2/7–positive cells are enriched in chemotherapy-resistant cells in both basal-like and luminal mammary tumors treated with cisplatin, and TcdB^FBD^ synergizes strongly with cisplatin in inhibiting both tumor types. These data demonstrate the therapeutic value of narrow-spectrum Wnt signaling inhibitor in treating breast cancers.

## Abstract 2

Wnt signaling is transmitted via ten homologous Frizzled receptors (FZD1-10) in humans, complicating the therapeutic use of inhibitors due to side-effects. This study shows that a bacterial toxin fragment can be used as a tool to specifically inhibit Wnt signaling mediated by a subset of Frizzed receptors (FZD1, 2, 7), with potential for cancer treatment.

## Introduction

Breast cancers are heterogeneous and different subtypes require distinct treatments [1,2]. Targeted therapy in breast cancer is most successful when subtype-specific key pathways that drive cancer cell growth are defined and serve as therapeutic targets. Among breast cancer subtypes, estrogen receptor (ER)^+^ luminal breast cancers are treated by endocrine therapy (e.g., aromatase inhibitor, tamoxifen) that targets the ER signaling pathway, whereas breast cancers with HER2 overexpression can be targeted by Trastuzumab (Herceptin), a monoclonal antibody that blocks HER2 signaling. Basal-like breast cancer largely overlaps with triple-negative breast cancer, which lacks ER and progesterone receptor (PR) expression and HER2 overexpression [3]. Basal-like/triple-negative breast cancers lack clear driver mutations, as evident from recent sequencing studies [4]. Thus, treatment of these breast cancers relies on standard chemotherapy, with the worst prognosis among all breast cancer subtypes [2]. In addition, even among luminal breast cancers, those belonging to the luminal B subtype have high proliferation index and often do not respond to endocrine therapy well; thus, they are treated by chemotherapy as well [5]. Chemotherapy can eliminate the bulk of cancer cells, but inevitably therapy-resistant cancer cells emerge, which typically possess stem cell-like properties [6,7]. In order to eliminate these cells, it is important to define key programs that sustain their stemness so that therapeutic approaches can be designed to target them.

Wnt/β-catenin signaling plays key roles in stem cell self-renewal and injury repair [8]. Mutations in components of the Wnt pathways are well-established as a dominant causal factor in colorectal cancer and many other solid tumors [8,9]. The therapeutic value of targeting Wnt signaling in these tumors are well-established using numerous animal models and by a number of broad-spectrum pan-Wnt signaling inhibitors [8–10]. Up-regulation of Wnt signaling without any mutations in the pathways has also been broadly implicated in cancer development, epithelial–mesenchymal transition (EMT), metastasis, chemotherapy-resistance, and immune escape of a broad range of human cancers [9,11], possibly reflecting a universal requirement of Wnt signaling in maintaining the stemness of cancer cells. For example, Wnt pathway activation has been previously reported to be enriched in basal-like breast cancer and predicts poor outcome [12], and activation of Wnt signaling (without mutations in Wnt pathways) is observed in >50% of human breast cancer cases and is linked to reduced overall survival [9]. This broad range of tumors potentially can benefit from Wnt signaling inhibition, but the side effect associated with pan-Wnt inhibition often diminishes the therapeutic value [13,14].

Wnt and its receptors, the 7-pass transmembrane protein Frizzled (FZDs), are a large family [8,15]. One way to minimize side effects is to target the specific Wnt-FZD pair(s) enriched in cancer cells. However, such narrow-spectrum inhibitors are difficult to develop due to high degrees of homology among Wnt-FZD members. There are also doubts on whether inhibiting a subgroup of Wnt-FZD pairs is sufficient to achieve any therapeutic effects in vivo.

There are 10 FZD members in humans, divided into 4 subgroups (FZD1/2/7, 5/8, 3/6, 4/9/10) [15]. They contain only 1 relatively small extracellular domain on their N-termini, designated cysteine-rich domain (CRD, approximately 120 to 150 residues), which serves as the binding site for Wnt [15]. CRDs are highly conserved across all mammals, and therefore, it has been difficult to generate specific antibodies against CRDs by immunization in animals. Instead, antibodies targeting specific CRDs including FZD5-CRD and FZD7-CRD have been previously generated and validated by screening various antibody libraries in vitro [16–19]. The most advanced Wnt-signaling inhibition antibody, OMP-18R5 (developed by OncoMed), which was identified through in vitro phage-display approach and bind 5 FZDs across 2 subgroups (FZD1/2/7 and FZD5/8), went through Phase II clinical trial, but its development has been suspended due to side effects including loss of bone density in patients [9,10,20]. Bone density loss is also a primary side effect associated with an Fc fusion protein containing FZD8-CRD [13,21].

FZD1/2/7 form a subgroup with nearly identical CRDs within the FZD family and FZD7 has been previously shown to be associated with triple-negative breast cancer and targeting FZD7 may reduce growth of breast cancer cells [22–25]. Here, we have developed and evaluated a specific inhibitor targeting Wnt-FZD1/2/7 signaling utilizing a fragment of *C*. *difficile* toxin B (TcdB) that binds to FZD1/2/7 [26,27]. Our previous work established that TcdB recognizes FZD1/2/7 subgroup as its major receptors. This recognition is highly specific as only expression of FZD1/2/7 in cells mediated binding of TcdB, but not any other FZD members [26]. We have also solved the structure of a TcdB fragment containing FZD-binding domain (amino acid residues 1285–1804, designated TcdB^FBD^) bound to FZD2-CRD, revealing that TcdB^FBD^ effectively blocks Wnt signaling by targeting a region in CRD that is critical for docking of the palmitate in Wnt [27]. All Wnts are modified by lipidation through the addition of a palmitoleic acid (PAM) to a conserved serine, which is essential for their secretion and binding to FZDs. Binding of TcdB^FBD^ prevents docking of the Wnt PAM into a hydrophobic groove in CRDs. Key residues for TcdB^FBD^ interactions are conserved in CRD1, 2, and 7, but varies in other FZD members, which are the reasons for the selective high-affinity binding of TcdB^FBD^ to CRD1/2/7 [27,28].

TcdB is a large protein (approximately 270 kDa) and recognizes multiple receptors via different domains. Previous studies have demonstrated that TcdB^FBD^ region does not recognize any other potential receptors for TcdB including chondroitin sulfate proteoglycan 4 (CSPG4) [26,29,30], poliovirus receptor-related 3 (PVRL3) [31], and LDL receptor–related protein 1 (LRP1) [32]. Furthermore, it has been shown binding of TcdB^FBD^ to FZD1/2/7 inhibits Wnt signaling mediated by these FZDs [26], demonstrating that TcdB^FBD^ can serve as a highly specific narrow-spectrum Wnt signaling inhibitor against FZD1/2/7.

Taking advantage of this specific inhibitor and utilizing several breast cancer mouse models including a novel one that we recently established, we demonstrate that TcdB^FBD^ suppresses tumor growth and cancer stem cell activities and synergizes with chemotherapy agent cisplatin in vivo, without side effects on bone density. These findings demonstrate both the feasibility and therapeutic value for narrow-spectrum Wnt signaling inhibitors for treating a broad range of breast cancers.

## Results

### FZD7 is the major FZD receptor expressed in basal-like breast cancer

To identify the specific subgroups of FZDs expressed in basal-like breast cancer, we examined expression of all 10 FZD members in human breast cancers and murine models. In publicly available human breast cancer expression data, *FZD5*, *6*, *7*, and *9* are expressed at higher levels in basal-like breast cancer cases than other subtypes (S1 Fig). Importantly, in both the METABRIC and TCGA breast cancer datasets, *FZD7* consistently exhibits higher mRNA levels than the other 3 *FZD*s in basal-like breast cancers (S2 and S3 Figs) [33–35]. In a microarray dataset for various breast cancer mouse models [36], *Fzd7* and *Fzd6* were highly expressed in *C3(1)-Tag* and *Trp53*-null ER-negative mammary tumors (S4 Fig), both of which represent mouse models for human basal-like/triple-negative breast cancers [37]. *Fzd7* was also highly expressed in 2 out of 4 *Trp53*-null ER-positive mammary tumors but exhibited medium/low expression levels in *Brca1* knockout (KO)/*Trp53*-wild type (WT) tumors (S4 Fig), raising a possibility of a potential role of p53-loss in *Fzd7* up-regulation.

To further examine Wnt signaling in murine breast cancer models, we focused on the above-described basal-like model *C3(1)-Tag* and the luminal B breast cancer model *MMTV-PyMT* [37], which exhibit high and medium/low levels of *Fzd7* expression, respectively (S4 Fig). We also included a novel basal-like breast cancer mouse model we established recently with loss of *Trp53* and *Brca1* [38]. This model is based on intraductal injection of a Cre-expressing adenovirus under the control of the keratin 8 (*Krt8*) promoter (*Ad-K8-Cre* [39]) to floxed *Trp53* and *Brca1* female mice carrying the conditional *Rosa26-LSL-EYFP* (*R26Y*) reporter (*Trp53*^*L/L*^;*Brca1*^*L/L*^*;R26Y*) (S5A and S5B Fig). This approach somatically inactivates *Brca1* and *Trp53* in luminal mammary epithelial cells, including luminal progenitors (LPs), which are believed to serve as the cellular origin of *BRCA1*-associated basal-like breast cancer [40–42]; we showed by single-cell analysis in this model that LPs were the target luminal mammary epithelial subset that underwent expansion, eventually leading to development of mammary tumors that closely resemble the human basal-like breast cancer subtype [38]. By immunofluorescence (IF) staining, we confirmed that both p53/BRCA1-deficient and *C3(1)-Tag* (but not *MMTV-PyMT*) mammary tumors contained many tumor cells expressing the basal marker, keratin 5 (K5), whereas all 3 tumor types were negative for ERɑ expression (S6 Fig). Of note, these 3 models represent mammary tumor types that are not good candidates for hormone or anti-HER2 therapies but can be treated by standard chemotherapy [43–46].

**Fig 1:**
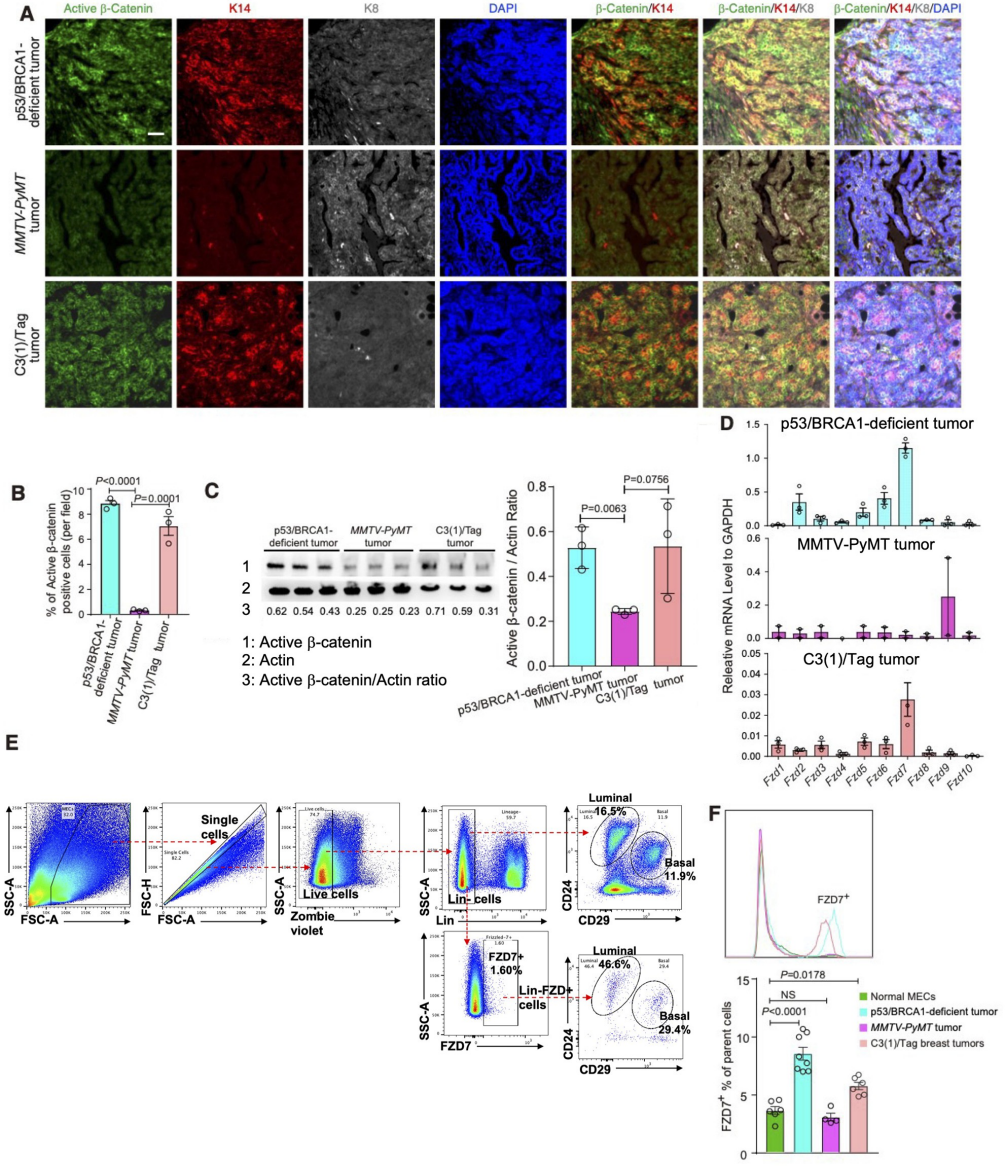
Wnt/FZD7 signaling is active in basal-like mammary tumors. **(A)** Representative immunostaining images detecting the levels of active β-catenin (non-phosphorylated form), K14, K8 in tumor tissues from *Trp53/Brca1*(p53/BRCA1)-deficient, *MMTV-PyMT* and *C3(1)-Tag* breast cancer mouse models. DAPI staining marks the cell nucleus. Scale bar = 50 μm. **(B)** Quantitation of active β-catenin positive cells in (A). **(C)** Immunoblot analysis of active β-catenin expression in mammary tumors from models described in (A and B). Actin serves as a loading control. **(D)** qRT-PCR analysis of expression of *Fzd* family genes in p53/BRCA1-deficient (*n* = 3), *C3(1)-Tag* (*n* = 3), and *MMTV-PyMT* (*n* = 2) tumors; error bars indicate mean ± SEM. **(E)** Upper plots: FACS analysis using antibodies against CD24 and CD29 to differentiate basal versus luminal normal MECs in female mice; lower plots: FACS analysis using a FZD7-specific antibody identified FZD7^+^ in Lin- (negative for lineage markers, CD45, CD31, TER119) cells and in luminal and basal MEC populations. **(F)** The percentages of FZD7^+^ cells in p53/BRCA1-deficient, *C3(1)-Tag* and *MMTV-PyMT* tumors, as well as in normal mouse mammary glands, were analyzed by FACS. The FZD7^+^ peaks are marked and compared in the upper panel and quantified in the lower panel (the *P* values for p53/BRCA1-deficient, *C3(1)-Tag* and *MMTV-PyMT* models were <0.0001, 0.8556, 0.0178, respectively, compared to normal mouse mammary glands). The representative FACS plots are shown in S5C Fig. Numerical values are in S1 Data. FACS, fluorescence-activated cell sorting; MEC, mammary epithelial cell; qRT-PCR, quantitative real-time PCR.

We first analyzed activation of Wnt signaling in these tumors by assessing their levels of active β-catenin (i.e., non-phosphorylated β-Catenin). Both p53/BRCA1-deficient tumors and *C3(1)-Tag* tumors exhibited higher levels of active β-catenin than that of *MMTV-PyMT* tumors, based on IF staining and immunoblot analysis (Fig 1A–1C and S7 Fig). Next, we measured expression levels of all *Fzd* genes in these mammary tumors by quantitative real-time (qRT)-PCR and found that only *Fzd7* was expressed at higher levels in both p53/BRCA1-deficient and *C3(1)-Tag* tumors, but not in *MMTV-PyMT* tumors, than all other *Fzd* genes (Fig 1D).

We then analyzed normal mammary gland tissues by fluorescence-activated cell sorting (FACS) with an anti-FZD7 antibody, which detected approximately 1% to 5% of lineage-negative (Lin^-^) live cells as FZD7-positive (FZD7^+^) (Fig 1E). Further analysis using CD24 and CD29 as markers revealed that FZD7^+^ cells include both luminal and basal mammary epithelial cells (Fig 1E). Of note, *FZD7* are also expressed in the luminal (including both the LP and hormone receptor (HR)^+^ luminal subsets) and basal epithelial populations in the human breast (S8 Fig). Compared to the normal mammary tissues, *MMTV-PyMT* tumor tissues contains similar levels of FZD7^+^ cells (both <5%), whereas both *C3(1)-Tag* and p53/BRCA1-deficient basal-like tumors, particularly the latter, contained elevated levels of FZD7^+^ cells (both >5%) (Fig 1F and S5C Fig). Together, these data suggest that FZD7^+^ cells are enriched in basal-like breast tumor models.

### TcdB^FBD^ inhibits FZD1/2/7-mediated Wnt signaling in human breast cancer cell lines

**Fig 2:**
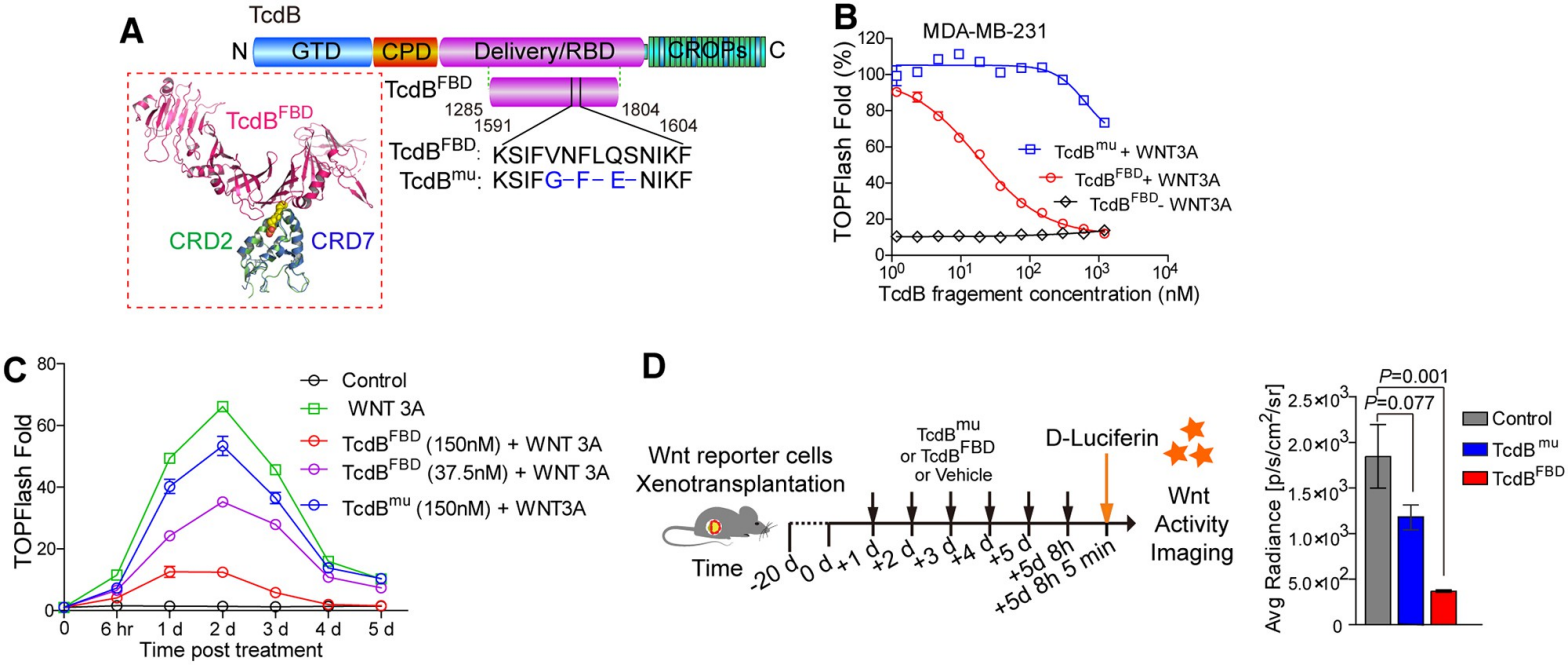
TcdB^FBD^ inhibits FZD1/2/7-mediated Wnt signaling. **(A)** Schematic diagrams showing the domain structures of TcdB and the 2 short fragments derived from TcdB (TcdB^FBD^ and TcdB^mu^) used in this study. GTD, glucosyltransferase domain; CPD, cysteine protease domain; Delivery/RBD, membrane translocation and receptor-binding domain; CROPs, combined repetitive oligopeptides domain. The structural model of TcdB^FBD^-CRD7 complex shown is modeled based on the crystal structure of TcdB^FBD^-CRD2 (PDB code: 6C0B) and CRD7 (PDB code:5T44). TcdB^FBD^, CRD2, and CRD7 are colored pink, green, and blue, respectively. CRD, cysteine-rich domain. **(B)** TcdB^FBD^ blocked WNT3A-mediated signaling in MDA-MB-231 cells in a dose-dependent manner, whereas TcdB^mu^ showed no inhibition at nanomolar concentrations. Wnt signaling activity was analyzed using the TOPFLASH/TK-Renilla (TK/RL) dual luciferase reporter assay (error bars indicate mean ± SEM, 3 independent experiments). **(C)** Wnt signaling activity in MDA-MB-231 cells was monitored using TK/RL assays over 5 days after induction by WNT3A conditioned medium with the indicated concentrations of TcdB^FBD^ or TcdB^mu^. Error bars indicate mean ± SEM, 3 independent experiments. **(D)** Nude mice were subcutaneously transplanted with TK/RL-transduced MDA-MB-231 cells and then treated at the indicated time point with TcdB^FBD^ or TcdB^mu^ (20 mg/kg of body weight) by intraperitoneal (i.p.) injection. D-Luciferin was injected 5 min before tumor tissues were isolated and the luciferase activity in tumor tissues was then measured ex vivo and quantified (error bars indicate mean ± SEM, *n* = 4–5 tumors). Numerical values are in S1 Data.

To evaluate whether FZD7 may serve as a therapeutic target, we took advantage of available TcdB^FBD^ (Fig 2A) [26,27]. We first tested the ability of TcdB^FBD^ to inhibit Wnt signaling in a human triple-negative breast cancer cell line MDA-MB-231 using a well-established TOPFLASH/TK-Renilla dual luciferase reporter assay. It has been previously reported that FZD7 is up-regulated in MDA-MB-231 [22], and qRT-PCR analysis confirmed that FZD1/2/7 are expressed at higher levels than other subgroups of FZDs in this cell line (S9A Fig). Wnt signaling in cells was stimulated using conditioned medium containing WNT3A. Nanomolar levels of TcdB^FBD^ inhibited WNT3A-mediated signaling in a dose-dependent manner (Fig 2B). This inhibitory effect sustained for over 72 h with a single exposure to TcdB^FBD^ in the medium (Fig 2C). As a control, a mutant form of TcdB (TcdB^mu^) that could no longer bind FZD1/2/7, constructed by replacing the key CRD-binding residues in TcdB^FBD^ with the corresponding residues in *C*. *difficile* toxin A that does not use FZDs as receptors (^1595^VNFLQS changed to ^1596^GFE, Fig 2A) [27,47], showed no inhibition of Wnt signaling at nanomolar concentrations (Fig 2B and 2C). TcdB^FBD^ did not affect viability of MDA-MB-231 and a few other human cell lines (U2OS, 293T, and MCF7), confirming that this toxin fragment does not have general toxicity to cells (S9B Fig).

To further evaluate the ability of TcdB^FBD^ to inhibit Wnt signaling in vivo, we injected MDA-MB-231 cells with an integrated TOPFLASH reporter subcutaneously into immunodeficient athymic nude mice, which resulted in tumor growth. Recombinantly purified TcdB^FBD^ was subsequently injected intraperitoneally (i.p.) to these mice at a dose of 20 mg/kg once per day for 6 times. To image the Wnt signaling activity, D-luciferin was injected 5 min (i.p.) in vivo before tumors were dissected out, and their luminescence signals were then measured ex vivo. Tumors from TcdB^FBD^-treated mice showed approximately 5-fold reduced signals compared with tumors from vehicle-treated control groups and TcdB^mu^-treated groups (Fig 2D). This experiment demonstrated that TcdB^FBD^ inhibits Wnt signaling in vivo.

We next evaluated the specificity of TcdB^FBD^ for targeting FZD1/2/7, but not other closely related FZDs (e.g., FZD5). Two pancreatic cancer cell lines, PaTu8988s and HPAF-II, are known to express high levels of FZD5 [48]. They are sensitive to small molecule pan-Wnt signaling inhibitor, LGK974, which targets the *O*-acyltransferase Porcupine required for palmitoylation of all Wnts [49]. While LGK974 treatment (at a concentration of 100 nM) inhibited clonogenic growth as well as the sphere formation ability of these 2 cell lines, TcdB^FBD^ treatment at a similar concentration (150 nM) exhibited no growth inhibitory effect on them in both assays (S9C–S9E Fig). Furthermore, we assessed the level of Wnt signaling in these cells by examining the amount of activated β-catenin in cell lysates (S9F and S9G Fig). While LGK974 treatment reduced activated β-catenin in cells, TcdB^FBD^ treatment showed no reduction, confirming that TcdB^FBD^ does not reduce Wnt signaling levels in these 2 pancreatic cancer cell lines (S9F and S9G Fig).

### TcdB^FBD^ inhibits growth of FZD7^+^ mammary tumors

**Fig 3:**
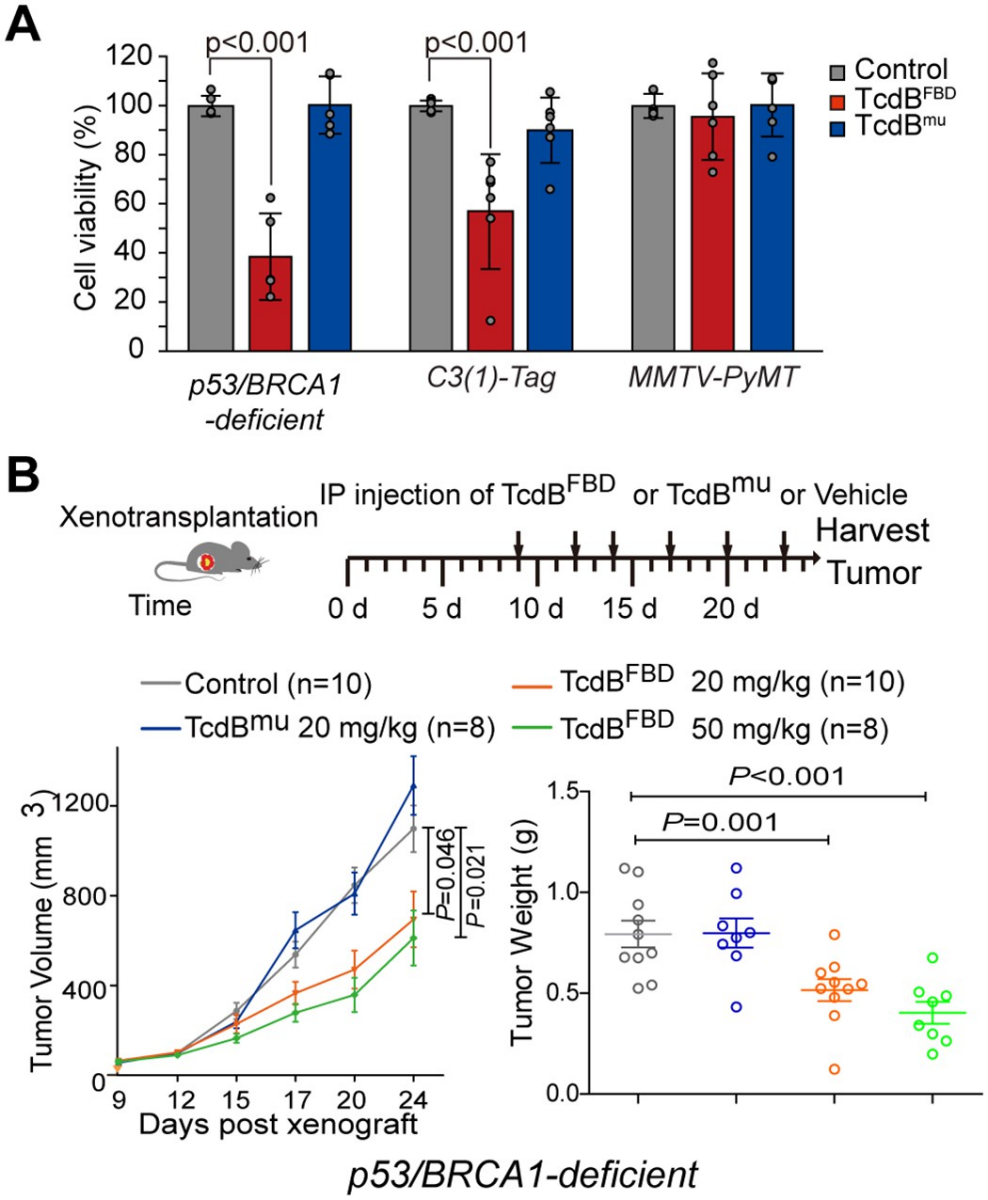
TcdB^FBD^ inhibits growth of basal-like mammary tumors. **(A)** Organoids were seeded in a 48-well plate. TcdB^FBD^ (150 nM) or TcdB^mu^ (150 nM) were added to the culture medium once per day. PBS was used as a vehicle control. After 5 days treatment, the viability of organoids was assessed using the ATP release assay. Error bars indicate mean ± SD; *N* = 5 for p53/BRCA1-deficient organoid; *N* = 6 for C3(1)-Tag and MMTV-PyMT organoid; data were considered significant when *p*-value < 0.01 (Student’s *t* test). **(B)** Mice were subcutaneously transplanted with p53/BRCA1-deficient mammary tumor organoid cells (10,000 cells per mice) and treated for the indicated period when tumor volumes reached 50 mm^3^ (left panel). The averaged tumor volumes over time were measured and plotted (middle panel). Tumor tissues were harvested on day 24 and weighted (right panel). Error bars represent SEM of 8–10 independently injected mice. Numerical values are in S1 Data.

To test whether TcdB^FBD^ could affect mammary tumors with FZD7 expression, we first treated tumor organoids derived from the above-described murine models with TcdB^FBD^ or TcdB^mu^. Treatment with TcdB^FBD^, but not TcdB^mu^, reduced the viability of organoids formed from the p53/BRCA1-deficient or *C3(1)-Tag* tumor cells; in contrast, TcdB^FBD^ treatment did not affect organoids formed from *MMTV-PyMT* luminal tumor organoids (Fig 3A). To further determine the specificity of TcdB^FBD^ in inhibiting Wnt signaling, we used CHIR99021, a small-molecule inhibitor of glycogen synthase kinase-3 (GSK3), which activates Wnt/β-catenin signaling downstream of FZDs. We found that the growth inhibition of p53/BRCA1-deficient tumor organoids by TcdB^FBD^ was rescued by CHIR99021 (S10A and S10B Fig), suggesting that TcdB^FBD^ acts on tumor organoids by suppressing Wnt signaling.

Our previous study showed that the mutated mammary epithelial cells underwent luminal to basal/mesenchymal cell fate change in p53/BRCA1-deficient breast cancer mouse model [38]. We thus examined expression levels of several EMT-related and Wnt signaling-related genes upon TcdB^FBD^ treatment. We found TcdB^FBD^-treated p53/BRCA1-deficient tumor organoids exhibited reduced expression of Wnt signaling-related genes (e.g., *Axin2*, *Rnf43*) and EMT-related genes (e.g., *Vim*, *Zeb1*) compared with the control and TcdB^mu^-treated organoids (S11A Fig).

Next, we utilized TcdB^FBD^ to evaluate whether inhibiting FZD7-mediated Wnt signaling may offer any therapeutic benefits in vivo. Subcutaneous injection of p53/BRCA1-deficient tumor organoid cells (1 × 10^4^ cells) into nude mice resulted in robust tumor growth. When tumors reached approximately 50 mm^3^, TcdB^FBD^ was injected i.p. into the recipient mice. TcdB^FBD^ administration at a dose of 20 or 50 mg/kg with the intervals indicated in Fig 3B, but not that of TcdB^mu^, attenuated tumor growth, although the effect was modest (Fig 3B). At molecular levels, genes related to Wnt signaling and EMT were down-regulated in tumors from TcdB^FBD^-treated mice, compared with those in vehicle or TcdB^mu^-treated groups (S11B Fig). Consistently, expression of lymphocyte enhancer-binding factor 1 (LEF1), a representative Wnt signaling effector, was reduced at the protein level (S11C Fig).

To further evaluate whether TcdB^FBD^ could attenuate growth of human breast cancer cells, we took advantage of a recently established biobank of over 100 primary and metastatic human breast cancer organoid lines [50]. We chose 2 organoid lines from this biobank, 74T and 86T, which represent a luminal and a basal-like breast cancer line with low and high level of a BRCA1-deficiency signature (i.e., signature 3), respectively [50]. The luminal organoid line 74T was insensitive to TcdB^FBD^ treatment (S12A Fig). In contrast, growth of the basal-like line 86T organoids was attenuated by TcdB^FBD^ (S12B Fig). Similar to the p53/BRCA1-deficient xenograft model, the inhibitory effect is rather modest and lacks a dose-dependency for unknown reasons. Nevertheless, these findings suggest that selective inhibition of FZD7-mediated signaling is sufficient to exhibit an inhibitory effect on growth of tumor cells, although Wnt signaling is not likely a driving force but rather one of the contributing factors in tumorigenesis of human breast cancers.

### TcdB^FBD^ treatment does not affect the intestine and bones

**Fig 4:**
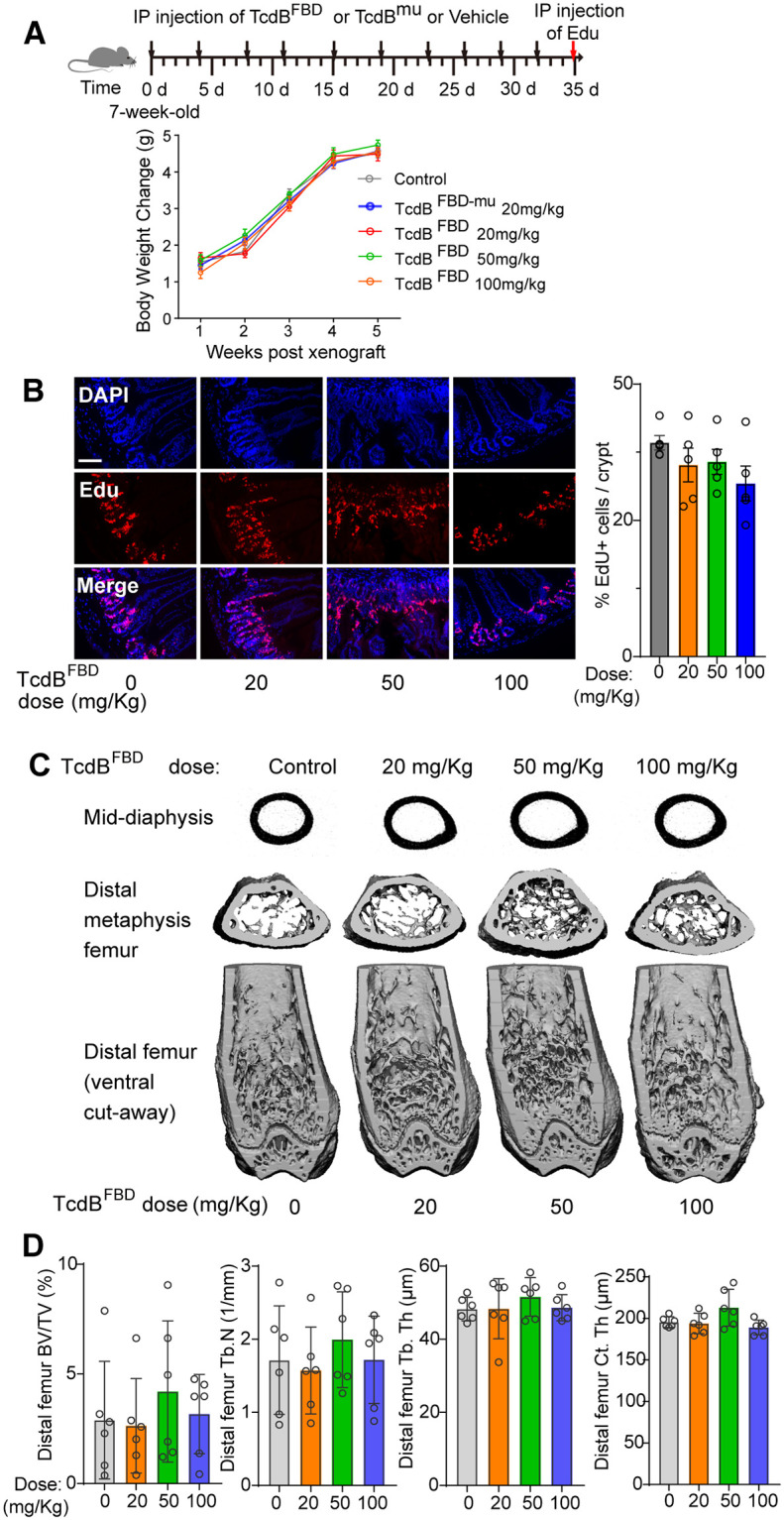
TcdB^FBD^ treatment in vivo does not affect the intestine and bones. **(A)** Six weeks old female nude mice (Hsd: Athymic Nude-*Foxn1*^*nu*^) were injected (i.p.) with the TcdB^FBD^ (20, 50, or 100 mg/kg) or TcdB^mu^ (20 mg/kg) twice a week for 5 weeks. Their body weight gains were monitored and plotted. Error bars indicate mean ± SEM, *n* = 8–10 mice. **(B)** Mice were treated with TcdB^FBD^ as described in panel A. EdU was injected (i.p., 100 mg/kg) 12 h before euthanization. Intestine tissues were harvested, fixed, and analyzed. The representative images were shown in the left panel and quantification of the percentage of EdU-positive cells per crypt was plotted in the right panel. Scale bar = 200 μm. *P* = 0.28. **(C)** Mice were treated with TcdB^FBD^ as described in panel A and their right femur bones were extracted, fixed, and subjected to micro-computed tomography (μCT) analysis. Representative μCT reconstructions of the midshaft femur cortical bone (upper row), distal femur metaphyseal bone (middle row), and entire distal femur with the ventral half of the femur digitally removed to reveal the cancellous compartment (lower row) are shown. N = 6 /dose group. **(D)** Quantification of the μCT analysis described in panel C for trabecular bone volume fraction (BV/TV), trabecular number (Tb.N), trabecular thickness (Tb.Th), and cortical area (Ct.Ar), *P* = 0.67, 0.63. 0.68, 0.67, respectively. Numerical values are in S1 Data.

We next analyzed whether inhibiting FZD7-mediated signaling by TcdB^FBD^ at therapeutically effective doses is tolerated in Wnt sensitive tissues such as the intestine and bones. TcdB^FBD^ was injected into mice at 20, 50, or 100 mg/kg twice a week for 5 weeks. These mice showed similar weight gains comparable with the control mice (Fig 4A). To examine the potential impact on the intestine, we injected (i.p.) EdU, which incorporates into replicating DNAs and marks proliferating cells, at the end of the fifth week. The intestine epithelium constantly turns over and newly generated cells are produced from stem cells located the bottom of the crypt region. Wnt signaling is a key pathway regulating intestinal stem cells and a reduction in EdU incorporation would reflect an inhibition on stem cell activity [51,52]. The intestinal tissues were dissected out and EdU incorporation was measured. As shown in Fig 4B, TcdB^FBD^ treatment at 20 and 50 mg/kg levels did not reduce EdU levels in the intestinal tissues. There appears to be a slight reduction at 100 mg/kg, but it did not reach statistical significance.

Clinical trials with OMP-18R5, which blocks FZD1/2/7 and 5/8, revealed that bone density loss is the single most critical side effect in humans [13]. Consistently, mice treated with pan-Wnt signaling inhibitors (Porcupine inhibitors LGK974 and ETC-1922159) exhibited loss of bone volume and density even at doses <10 mg/kg [53]. We thus focused our analysis on bone mass and architecture utilizing micro-computed tomography (μCT) technology in mice treated with TcdB^FBD^ at 20, 50, and 100 mg/kg doses for 5 weeks. No differences were observed in cancellous or cortical microstructure in the femur (Fig 4C). Bone volume fraction (BV/TV), trabecular number (Tb.N), and trabecular thickness (Tb.Th) in the distal femur metaphysis were not affected by TcdB^FBD^, neither were cortical bone parameters, e.g., cortical thickness (Ct.Th) (Fig 4D). These data suggest that TcdB^FBD^ may have a higher therapeutic safety window compared with LGK974, although additional pharmacokinetic studies would be required to fully examine the effective and safety doses of TcdB^FBD^.

### TcdB^FBD^ inhibits tumor sphere/organoid-forming cells

**Fig 5:**
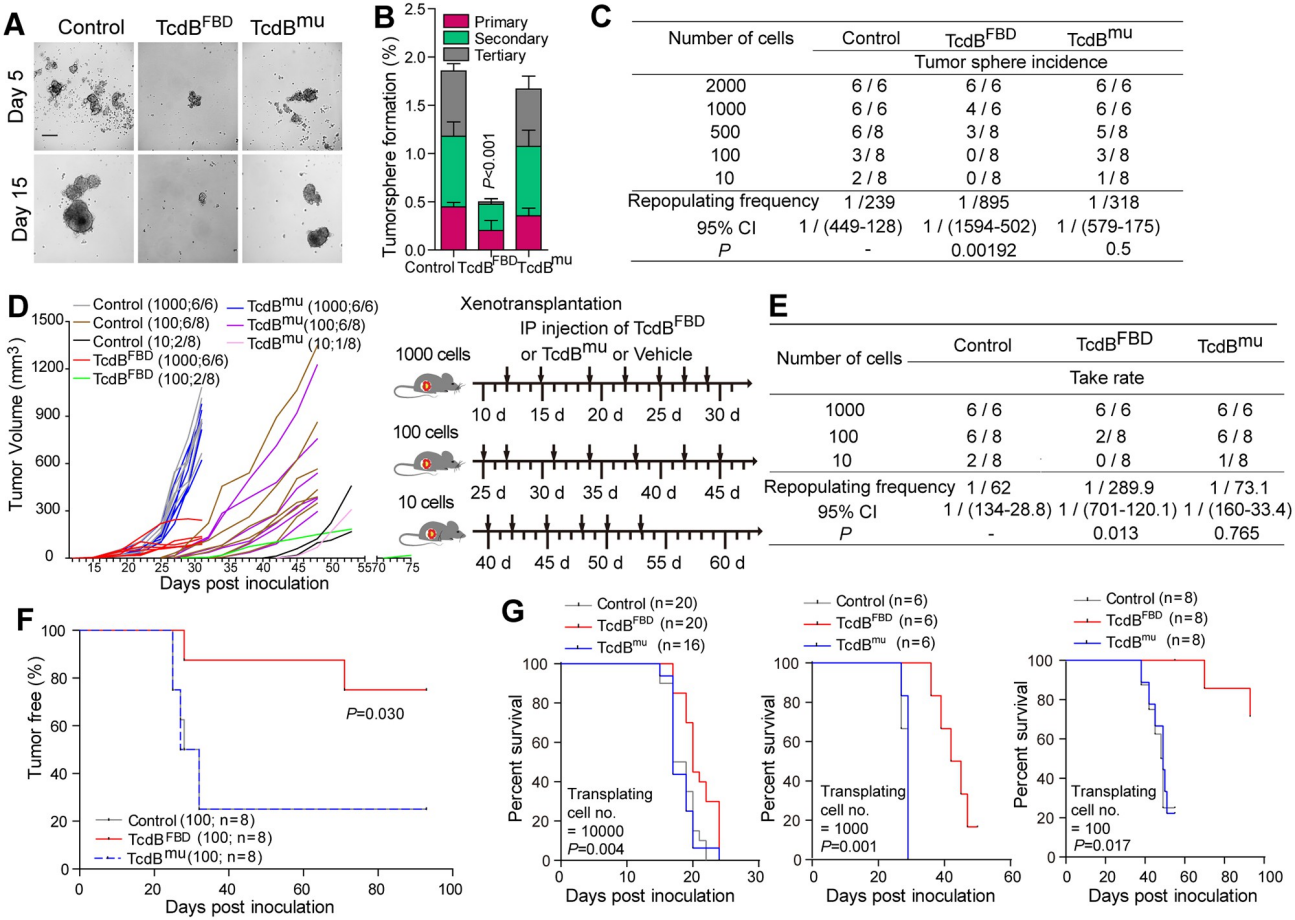
TcdB^FBD^ inhibits tumorigenic potential of basal-like mammary tumor cells. **(A)** Cells dissociated from p53/BRCA1-deficient mammary tumor tissues were resuspended and plated into round-bottom 96-well ultralow attachment plates in the sphere culture medium containing with 150 nM TcdB^FBD^, TcdB^mu^, or PBS (control). Representative images of tumorspheres formed at day 5 and day 15 in culture are shown. Scale bar = 200 μm. **(B)** The percent of tumorspheres were quantified following 3 passages of p53/BRCA1-deficient mammary tumor cells (5,000 cells/well for primary culture and 2,000 cells/well for secondary and tertiary culture) with 150 nM TcdB^FBD^ or TcdB^mu^ or PBS in the sphere culture medium. **(C)** Analysis of the sphere-forming capabilities of p53/BRCA1-deficient mammary tumor cells using the limiting dilution assay. Cells were seeded in the presence of 150 nM TcdB^FBD^ or TcdB^mu^ or PBS vehicle. Sphere formation was counted 10–15 days post-seeding. The frequency of sphere-forming cells (TICs) was calculated using the ELDA website (http://bioinf.wehi.edu.au/software/elda/index.html): Control = 1/239 (lower 449, upper 128); TcdB^FBD^ = 1/895 (lower 1,594, upper 502), *P* = 0.00192, compared with control, TcdB^mu^ = 1/318 (lower 579, upper 175). **(D)** Tumor organoid cells (10, 100, or 1,000 cells) derived from p53/BRCA1-deficient tumors were injected subcutaneously into nude mice. The growth of tumor was evaluated daily for 3 months. Once 1 tumor grows out in each group (10, 100, or 1,000 cells), the mice was divided into 3 groups, received one of the following treatments twice a week: PBS (150 μl/mice), endo-toxin free TcdB^FBD^ (20 mg/kg), or TcdB^mu^ (20 mg/kg). Values in brackets indicate the number of organoid cells, the number of tumors obtained versus the total injected mice number. **(E)** Analysis of the tumorigenesis of p53/BRCA1-deficient mammary tumor organoid cells using the limiting dilution assay as described in (D). Frequency of TICs: Control = 1/62 (lower 134, upper 28.8); TcdB^FBD^ = 1/289.9 (lower 701, upper 120.1), *P* = 0.0013, compared with control, TcdB^mu^ = 1/73.1 (lower 160, upper 33.4). **(F)** Tumor latency plotted as percentage of tumor-free mice implanted with 100 p53/BRCA1-deficient mammary tumor organoid cells following the indicated treatment. **(G)** Kaplan–Meier survival curves of nude mice bearing xenograft tumors treated as indicated. Numerical values are in S1 Data.

FACS analysis demonstrates that approximately 90% of TcdB^FBD^-bound cells from primary p53/BRCA1-deficient tumors were FZD7^+^ (S13A Fig), confirming that TcdB^FBD^ targeted FZD7^+^ cells in tumor tissues. As Wnt signaling plays key roles in maintaining stem cell activities, we next examined whether TcdB^FBD^ affected the activity of tumor sphere-forming cells in the p53/BRCA1-deficient tumor, utilizing the mammosphere/tumorsphere assay by culturing cancer cells in suspension [54,55]. Dissociated tumor cells from the p53/BRCA1-deficient model formed spheroids (i.e., tumorspheres) in suspension culture (S13B Fig). FACS analysis revealed that approximately 85.7% of cells forming tumorspheres are FZD7^+^ (S13C Fig). TcdB^FBD^ treatment greatly reduced sphere formation of p53/BRCA1-deficient mammary tumor cells (Fig 5A). After TcdB^FBD^ treatment, the percentage of tumorsphere formation in the first passage was 1.2-fold reduced compared to that of control, increased to 1.7-fold reduction at the second passage, and became even more profound in the third passage (26.0-fold reduction) (Fig 5B and S1 Table). These data indicate that TcdB^FBD^ inhibited the self-renewal potential of p53/BRCA1-deficient tumor cells. To further determine the effect of TcdB^FBD^, limiting dilution assay was employed by serial re-plating of p53/BRCA1-deficient tumor cells at various cell concentrations in the sphere culture. The frequency of sphere-forming cells, a surrogate for the frequency of tumor-initiating cells, was drastically reduced after TcdB^FBD^ treatment, dropping from 0.42% (1/239) to 0.11% (1/895) compared to control (*P* = 0.0019) (Fig 5C). Furthermore, the suppression of tumorsphere formation by TcdB^FBD^ could be rescued by CHIR99021 (S2 Table). Collectively, these data demonstrate that TcdB^FBD^ targeted tumor sphere-forming cells and suppressed their capability to generate tumorsphere in vitro.

To further assess the effect of TcdB^FBD^ on tumor-initiating cells in vivo, we performed the limiting dilution assay by transplanting approximately 10 to 1,000 p53/BRCA1-deficient tumor organoid cells to nude mice. FACS analysis confirmed that most of these tumor organoid cells were FZD7^+^ (approximately 81.7%, S13D and S13E Fig). TcdB^FBD^ or TcdB^mu^ treatment was administered at 20 mg/kg twice a week starting from day 5 after inoculation until day 53, when most mice had reached their endpoint (Fig 5D). Inoculating 1,000 organoid cells resulted in tumor formation in all mice within approximately 2 weeks. Reducing the number of organoid cells decreased the frequency in tumor formation, and a repopulating frequency can be calculated from a serial dilution of organoid cells (Fig 5E). TcdB^FBD^ treatment reduced this frequency by 3.68-fold from 1/62 in control mice to 1/289.9, whereas TcdB^mu^ treatment did not change the frequency (Fig 5E). Specifically, transplanting as few as 10 tumor organoid cells in the control or TcdB^mu^-treated group formed tumors 2 out of 8 times, whereas no tumor was detected in similarly transplanted mice treated with TcdB^FBD^ (Fig 5D and 5E). Furthermore, mice transplanted with 100 organoid cells and treated with TcdB^FBD^ exhibited a significantly longer tumor-free period than those received vehicle or TcdB^mu^ treatment (average tumor-free time 82.13 days versus 44.37 days, *P* = 0.030) (Fig 5F). Consistently, mice inoculated with 10,000, 1,000, or 100 p53/BRCA1-deficient tumor organoid cells and treated with TcdB^FBD^ survived 1.2 days (*P* = 0.014), 14.84 days (*P* = 0.001), or 22.88 days (*P* = 0.017) longer than those treated with vehicle or TcdB^mu^ (Fig 5G). Collectively, these results suggest that the inhibition of TcdB^FBD^ on tumor growth is mediated by suppressing tumor-initiating cells in vivo.

### TcdB^FBD^ synergizes with cisplatin in treating both basal-like and luminal breast cancers

**Fig 6:**
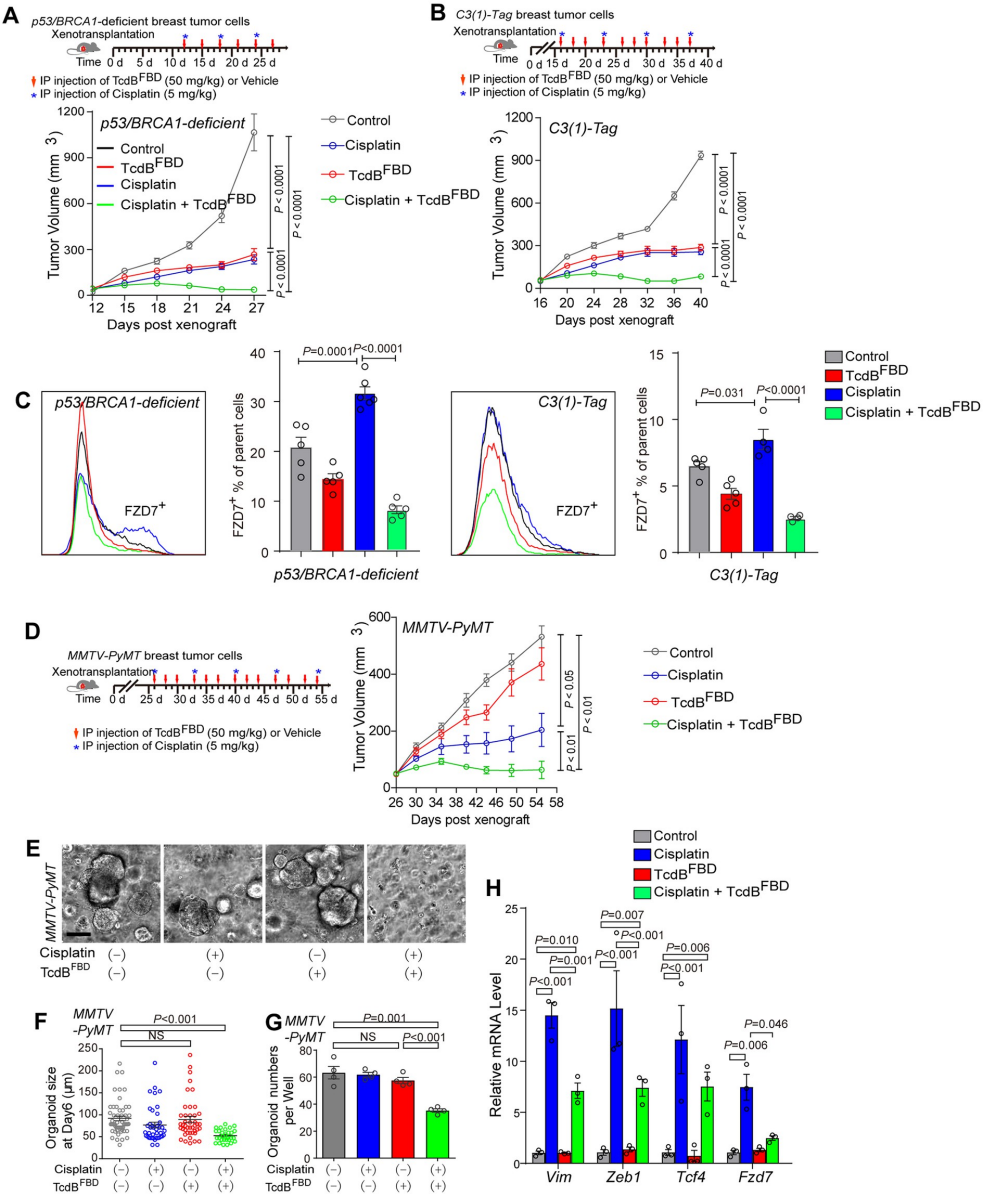
TcdB^FBD^ inhibits cisplatin-resistant mammary tumor cells. **(A, B)** Tumor organoid cells from p53/BRAC1-deficient tumor model (A) or *C3(1)-Tag* model (B) were injected into nude mice, and mice were then treated with TcdB^FBD^ alone, cisplatin alone, or a combination of both TcdB^FBD^ and cisplatin as indicated. Tumor volumes were recorded and plotted over time. Error bars indicate mean ± SEM, *n* = 8 mice. **(C)** FACS analysis of FZD7^+^ cells in the indicated tumors from p53/BRCA1-deficient (*n* = 5) and *C3(1)-Tag* (*n* = 4) models; the FACS gating strategy is the same as those shown in S5C Fig. Error bars indicate mean ± SEM. **(D)** Tumor organoid cells from *MMTV-PyMT* model were injected into nude mice, and mice were then treated with TcdB^FBD^ alone, cisplatin alone, or a combination of both TcdB^FBD^ and cisplatin as indicated. Tumor volumes were recorded and plotted over time. Error bars indicate mean ± SEM, *n* = 6–8 mice. **(E)** Representative images of *MMTV-PyMT* mammary tumor organoids treated with TcdB^FBD^ alone (150 nM), cisplatin alone (0.2 μM), or a combination of cisplatin and TcdB^FBD^. Scale bar = 100 μm. **(F)** Quantitation of organoid sizes for indicated groups described in (E). **(G)** Quantitation of organoid numbers for indicated groups described in (E). **(H)** qRT-PCR analysis of EMT-related genes (*Vim*, *Zeb1*, *Tcf4*) and *Fzd7* genes in tumor cells from *MMTV-PyMT* model after treatment with TcdB^FBD^, cisplatin, or a combination of both (*P* < 0.01). Numerical values are in S1 Data. EMT, epithelial–mesenchymal transition; FACS, fluorescence-activated cell sorting; qRT-PCR, quantitative real-time PCR.

Even though TcdB^FBD^ exhibited efficacy in inhibiting growth of basal-like breast cancer via targeting tumor-initiating cells, it is unlikely to eliminate tumors as a single agent. We thus examined its therapeutic value in combination with a standard chemotherapy drug (e.g., cisplatin). Xenograft experiments with the same initial number of tumor organoid cells from the p53/BRCA1-deficient and *C3(1)-Tag* models were performed in nude mice. Mice were then treated with TcdB^FBD^ alone, cisplatin alone, or a combination of both agents following the schedule illustrated in Fig 6A and 6B. TcdB^FBD^ alone reduced tumor growth in both models and its efficacy is similar to treatment with cisplatin alone (Fig 6A and 6B). Combination treatment with both TcdB^FBD^ and cisplatin achieved the highest level of growth inhibition than either agent alone on tumors derived from p53/BRCA1-deficient and *C3(1)-Tag* models, demonstrating a strong synergistic effect (Fig 6A and 6B). To further confirm the synergistic effect, we also examined the sensitivity of tumor organoids of these 2 basal-like cancer models. A low concentration of cisplatin (0.2 μM) did not reduce the size or number of tumor organoids, whereas combining this level of cisplatin with TcdB^FBD^ reduced both size and number and organoids further than treatment with TcdB^FBD^ alone (S14 Fig). These results suggest that targeting FZD7-mediated signaling may overcome the resistance of tumor cells to cisplatin, or cisplatin treatment may sensitize cells to FZD7-mediated inhibition by TcdB^FBD^. It is possible that a subpopulation of FZD7^+^ tumor cells is intrinsically more resistant to chemotherapy, or FZD7 is up-regulated in a subpopulation of cells that developed resistance. Indeed, FACS analysis of tumor cells revealed that the percentage of FZD7^+^ cells increased after treatment with cisplatin alone in both p53/BRCA1-deficient and *C3(1)-Tag* models, and co-administration of TcdB^FBD^ greatly reduced FZD7^+^ cells (Fig 6C).

We also tested the luminal tumor *MMTV-PyMT* model in xenograft models in vivo, which do not overexpress *Fzd7* and its organoids are resistant to TcdB^FBD^ in vitro (Figs 1D and 3A). As expected, TcdB^FBD^ alone did not affect tumor growth in this model in vivo (Fig 6D). Surprisingly, TcdB^FBD^ was able to synergize with cisplatin in this model (Fig 6D). Consistently, although neither a low concentration of cisplatin nor TcdB^FBD^ alone affected tumor organoids of *MMTV-PyMT* model, a combination of cisplatin and TcdB^FBD^ reduced the size and number of organoids (Fig 6E–6G). Thus, exposure to cisplatin may have turned the TcdB^FBD^-insensitive tumor into a sensitive one. These findings are consistent with our recent report that cisplatin treatment could lead to luminal to basal/mesenchymal cell fate changes, in part due to interstrand DNA crosslinks [38]. Indeed, treatment with cisplatin increased expression of basal/mesenchymal genes, as well as *Fzd7* in *MMTV-PyMT* tumors, whereas these changes were reduced in the presence of TcdB^FBD^ (Fig 6H), suggesting that cisplatin-induced cell fate change renders cells sensitive to the co-inhibition of FZD7-mediated Wnt signaling in luminal breast tumors.

## Discussion

Wnt signaling is a key pathway in maintaining the stemness of cells. Besides well-established cancer-causing mutations in Wnt pathways, recent studies have also suggested that Wnt signaling up-regulation contributes to many aspects of cancer development and therapy resistance, including EMT, metastasis, and resistance to chemotherapy and immunotherapy. Thus, Wnt signaling represents a major therapeutic target in cancer treatment [13]. Broadly inhibiting Wnt signaling inevitably results in side effects, limiting the therapeutic safety window. Inhibiting a subset of Wnt signaling may reduce the side effect and it needs to be done at the ligand-receptor level as this is the most diverged node in the pathway [15], but it has been difficult to develop inhibitors that can distinguish different FZD subfamily members. Furthermore, whether targeting only a subset of FZDs is going to be sufficient remains to be demonstrated in vivo. Here, we utilized the natural ability of a bacterial toxin to specifically recognize FZD1, 2, 7 subgroup and block FZD1/2/7-mediated Wnt signaling. We found that blocking FZD1/2/7-mediated Wnt signaling reduced growth of basal-like tumor models in vivo and also synergized with chemotherapy agent cisplatin in treating breast tumor models.

Chemotherapy remains to be the cornerstone of the first-line treatment for many tumors. Conventional chemotherapy is effective in controlling primary tumors, but often leads to eventual regeneration of chemo-resistant tumors and metastasis. Our findings that TcdB^FBD^ synergizes with cisplatin treatment add to the wealth of literatures suggesting that Wnt signaling plays a key role in the development/selection of treatment-resistant cells. Synergistic effect from Wnt signaling inhibition has also been reported between OMP-18R5 and the chemotherapy agent taxanes on a range of tumor models [10]. Future studies can focus on testing whether TcdB^FBD^ would also be effective in shrinking mammary tumors when combined with other types of chemotherapeutic agents (e.g., doxorubicin) and whether combined TcdB^FBD^ and cisplatin treatment could be applied to other cancer types (e.g., serous ovarian cancer, oral squamous cell carcinoma).

Up-regulation of Wnt signaling has been associated with the resistance to many common chemotherapy agents as well as radiation therapy [11]. The mechanistic link between Wnt signaling and therapy resistance remains to be fully established and is likely multifaceted [11]. For example, recent studies suggest that Wnt signaling enhances the DNA repair pathways in ovarian cancers [56]. In our study, it is likely that cisplatin treatment leads to an epithelial cell fate change toward a mesenchymal state [38], which may represent a dedifferentiation process that involves Wnt signaling. Lastly, Wnt signaling has been shown to contribute to immune invasion as well as systemic inflammation in the tumor microenvironment that drives cancer metastasis [57–60]. Whether TcdB^FBD^ may provide a synergistic effect with immunotherapy such as PD-1/PD-L1 antibodies remains to be explored.

Intestines and bones are 2 major tissues sensitive to disruptions in Wnt signaling pathways and are often examined when testing Wnt signaling pathway inhibitors. The finding that TcdB^FBD^ did not affect intestinal tissues is consistent with previous findings that the pan-Wnt inhibitors LGK974 did not cause any damage to the intestine until a dose much higher than the therapeutic dose [49]. These findings are also supported by clinical observations that intestinal damage is not a major side effect in clinical trials of OMP-18R5 antibody. On the other hand, bone density loss is the single most important side effect observed from clinical trials of OMP-18R5. Our analysis showed that bones were not affected in mice by TcdB^FBD^, which represents a major advantage over other existing pan-Wnt signaling inhibitors and neutralizing antibodies. This is possibly because both FZD7 and FZD5 are expressed in diaphyseal bone and potentially play redundant roles [61]. FZD5-mediated Wnt signaling in bone tissues might be sufficient to maintain bone density when FZD7 is selectively inhibited by TcdB^FBD^, whereas the neutralizing antibody OMP-18R5 inhibits both FZD1/2/7 and FZD5/8 subfamilies.

The specificity of TcdB^FBD^ for FZD1/2/7 allows us to establish this agent as an effective targeted therapy for FZD1/2/7^+^ and/or cisplatin-resistant mammary tumor cells. A major limitation of TcdB^FBD^ is that this is a bacterial protein that induces neutralizing antibodies within the body. Furthermore, prior exposure to TcdB during *C*. *difficile* infection could result in existing immunity and lowering the efficacy of TcdB^FBD^. The crystal structure of TcdB^FBD^-CRD complex showed that the toxin targets the lipid docking site in CRD. The sequence variations surrounding this lipid docking site across different FZDs underlies the reason for the selectivity of TcdB toward FZD1/2/7 [27,28]. These data suggest that this lipid docking site may serve as a promising therapeutic target for developing effective and selective Wnt signaling inhibitors and antibodies.

## Methods

### Ethics statement

All animal experiments were approved by the Institutional Animal Care and Use Committee (IACUC) of the Brigham and Women’s Hospital and Boston Children’s Hospital where these mice were housed (Protocol #20-09-4202R).

### Cloning, expression, and purification of recombinant proteins

The gene encoding TcdB^FBD^ (residues 1285–1804) was cloned into pET28a vector, with an N-terminus HA tag. Mutated TcdB^FBD^ variants (TcdB^mu^) were generated by two-step PCR and verified by DNA sequencing. TcdB^FBD^ and TcdB^mu^ were expressed in *E*. *coli* strain BL21-Star (DE3) (Invitrogen). Bacteria were cultured at 37 °C in LB medium containing kanamycin. The temperature was reduced to 16 °C when OD600 reached approximately 0.8. Expression was induced with 1 mM IPTG (isopropyl-b-D-thiogalactopyranoside) and continued at 16 °C overnight. Proteins were purified using Ni2+-NTA (nitrilotriacetic acid, Qiagen) affinity resins in a buffer containing 40 mM imidazole, 400 mM NaCl, and 50 mM Tris (pH 8.0). The proteins were eluted with a high-imidazole buffer (300 mM imidazole, 400 mM NaCl, and 50 mM Tris (pH 8.0)) and then dialyzed at 4 °C against a buffer containing 150 mM NaCl and 20 mM HEPES (pH 7.5). Proteins were further purified by MonoQ ion-exchange (20 mM Tris (pH 8.5)) and Superdex-200 size-exclusion chromatography (GE Healthcare, 20 mM Tris (pH 8.0), and 100 mM NaCl). Proteins were further incubated with Endotoxin Removal Resin (Thermo Scientific, # 88270) at 4 °C with gentle mixing for 1 h in columns, collected by centrifugation, further concentrated to approximately 10 mg/ml in PBS, and stocked in aliquots at −80 °C.

### Cell lines and constructs

MDA-MB-231 (# HTB-26), MCF7 (# HTB-22), U2OS (# HTB-96), 293T (#CRL-3216), HPAF-II (#CRL-1997), L cells (#CRL-2648), and L/WNT3A (#CRL-2647) cells were originally obtained from ATCC. PaTu8988s was generously provided by Stephane Angers (University of Toronto, Canada). The cells were cultured in DMEM medium supplemented with 10% fetal bovine serum, 10,000 I.U./ml penicillin, 50 μg/ml streptomycin (Invitrogen) in a humidified atmosphere containing 5% CO_2_ at 37 °C. Stable Wnt-reporter cells (MDA-MB-231-TK/RL, U2OS-TK/RL, 293T-TK/RL cells) were generated by lentiviral transduction of MDA-MB-231, U2OS, 293T cells with constructs expressing Renilla and firefly luciferases (7xTcf-FFluc, # 24308; RLUC—IRES–FLUC, # 45642, Addgene), followed by selection with 3 μg/ml puromycin and 50 μg/ml geneticin.

### Preparation of WNT3A-conditioned induction medium

WNT3A-conditioned induction medium was generated using L-WNT3A cells according to the manufacturer’s protocol. Conditioned medium from the corresponding L-cells was collected and used as a control. Briefly, the cells were grown in 10 ml of DMEM supplemented with 10% FBS for 4 days prior to collecting conditioned medium. To these cells, another 10 ml of fresh medium was added and cultured for 3 days to collect the second batch of conditioned medium. The 2 batches of conditioned media were mixed at a 1:1 ratio and filtered using 0.22 μm filter and stored at 4 °C until usage.

### Wnt signaling assay

The TOPFLASH/TK-Renilla dual luciferase reporter assay (# E1910, Promega) was used to detect Wnt signaling activities. Briefly, Wnt signaling activates expression of TOPFLASH luciferase reporter (firefly luciferase); Renilla luciferase serves as an internal control. To obtain Wnt signaling inhibition dose response curve for TcdB^FBD^ in Wnt reporter cells, MDA-MB-231- TK/RL, U2OS- TK/RL, 293T- TK/RL cells in 24-well plates were treated with a range of concentrations of TcdB^FBD^ with 10 doses in 2-fold dilution series in WNT3A-conditional medium for 6 h. GraphPad Prism software was used for graphing and EC50 calculations.

### Viability assays

MDA-MB-231, MCF7, U2OS, 293T cells were seeded at 3,000 to 5,000 cells per well in 96-well plates. Twenty-four h after seeding, cells were treated with TcdB^FBD^ in triplicates, with 10 doses in 2-fold dilution for 72 h, the MTT solution was added to the culture medium (500 μg/ml). After incubation at 37 °C for 3 h, followed by the addition of 200 μl of dimethyl sulfoxide (DMSO) to solubilize MTT. The absorbance at 562 nm was measured on a microplate reader. Normal cells without exposure to TcdB^FBD^ were considered 100% viable. The viability of organoids was assessed using the CellTiter-Glo luminescent cell viability assay (#G7570, Promega) according to the manufacturer’s protocol. Data were represented as mean ± SD from multiple biological replicates.

### Colony formation assay

HPAF-II and PaTu8988s cells were seeded in 24-well plates at a density of 2,000 cells per well and cultured in the culture medium added with 100 nM LGK974 or 150 nM TcdB^FBD^ or PBS vehicle control for 11 days and medium was refreshed every 3 days. Cells were washed by PBS, fixed by 4% paraformaldehyde for 15 min, stained with 0.5% crystal violet for 1 h, washed 3 times by ddH_2_O, and then photographed with a digital camera. The number of colonies was counted.

### Mouse models

The *C3(1)-Tag* transgenic mice (FVB-Tg(C3-1-TAg)cJeg/JegJ, Stock No: 013591), *MMTV-PyMT* (FVB/N-Tg(MMTV-PyMT)634Mul/J, Stock No: 002374), *Trp53*^*L*^ (B6.129P2-*Trp53*^*tm1Brn*^/J, Stock No: 008462), *Brca1*^*L*^ (STOCK *Brca1*^*tm1Aash*^*/*J, Stock No: 017835), *R26Y* (B6.129X1-*Gt(ROSA)26Sor*^*tm1(EYFP)Cos*^/J, Stock No: 006148) mice were obtained from The Jackson Laboratory. To target luminal mammary epithelial cells in *Trp53*^*L/L*^*;Brca1*^*L/L*^*;R26Y* adult female mice (2 to 3 months of age) were anaesthetized, and *Ad-K8-Cre* adenovirus (diluted in 0.1% bromophenol blue in DMEM) was introduced into ducts of the fourth mammary gland via intraductal injection [39]. Mice were then monitored via palpation or visual inspection weekly for tumor appearance. Once tumors were detected, animals were monitored 3 times a week for tumor development. For xenograft studies, 6 to 8 weeks old female nude mice (Hsd: Athymic Nude-*Foxn1*^*nu*^, 6903F) were purchased from Envigo RMS (Indianapolis, Indiana, United States of America).

### Tumor dissociation

Mammary tumors from the above mice were harvested, dissected and minced, and then incubated in digestion medium (2% penicillin/streptomycin, 0.1 mg/ml gentamicin, 0.6% nystatin, 2 mg/ml collagenase A, 0.096 mg/ml hyaluronidase in DMEM/F12) at 37 °C with shaking for 2 h. After digestion, the cells/tissues were treated sequentially with 0.25% trypsin/EDTA (37 °C, 2 min), 5 mg/ml dispase with DNaseI (0.1 mg/ml, Sigma, St. Louis, Missouri; 37 °C, 5 min), cold red blood cell (RBC) lysis buffer (2 to 3 min). Between each treatment step, cells/tissues were washed with PBS. After treatment with the RBC lysis buffer, cells/tissues were filtered through 40 μM cell strainer to obtain single-cell suspension.

### Organoid culture

A total of 2,000 single cells digested from the corresponding primary tumors were seeded in 20 μl Matrigel in a 48-well plate, cultured in 250 μl DMEM/F12 supplemented with 12 mM Hepes, 1% GlutaMAX, 1:50 B27, 0.21 μg/ml A83-01, 0.1 μg/ml EGF, 10 μM Y-27632, 100 ng/ml Noggin, and 0.6 μg/ml R-spondin1. Established clonal organoids were trypsinized using TrypLE (Thermo Fisher Scientific). TcdB^FBD^ or TcdB^mu^ (150 nM) was added to the culture medium every day; PBS was used as a vehicle control.

### Tumor implantation and evaluation

To establish xenograft tumors, a single p53/BRCA1-deficient tumor organoid cell suspension was harvested after trypsinization. The cells were resuspended in DMEM and Matrigel (V:V = 1:1) and then were injected into the flank of nude mice. Tumors could be observed 5 to 7 days after organoid cell inoculation (1 × 10^4^), tumor volumes were measured, and the mice were weighed twice weekly. Tumor volume was calculated using the formula: ½ (Length × Width^2^). When tumors reach approximately 50 mm^3^ (approximately 9 days post inoculation of 1 × 10^4^), mice were randomly divided into 4 groups (8 or 10 mice/group) and the mean tumor volumes of each group were similar. No mice were excluded during the treatment. Each group received one of the following treatments: PBS (150 μl/mice), endo-toxin free TcdB^FBD^ (20, 50, 100 mg/kg), or TcdB^mu^, once a day on day 9, 12, 14, 17, 20, and 23 post inoculation via intraperitoneal injection (i.p.). For combination treatment, each group received one of the following treatments: PBS (150 μl/mice), cisplatin (5 mg/kg) alone, or with endo-toxin free TcdB^FBD^ (20 mg/kg) once a day on the indicated day post inoculation via intraperitoneal injection (i.p.). Cisplatin was freshly prepared in PBS for in vivo administration at a final concentration of 5 mg/kg body weight.

### Bioluminescent assay

MDA-MB-231 cells that express integrated TOPFLASH were subcutaneously injected into immune-deficient athymic nude mice, when tumor length reached 10 mm, TcdB^FBD^ or TcdB^mu^ was injected at 20 mg/kg dose at indicated time. Five minutes before sacrifice, 100 mg/kg D-Luciferin was given (i.p.), tumors were dissected, put into 24-plate, and bioluminescent imaging were examined using a Xenogen IVIS-200 system (Xenogen). Images were analyzed by quantification of total photon flux of each tumor using Living Imaging Software. The p53/BRCA1-deficient tumor organoid cells were transduced by lentivirus with constructs 7xTcf-FFluc and subjected to the same analysis as described for MDA-MB-231 cells.

### TcdB^FBD^ in vivo toxicity assay or EdU staining

Six weeks old female nude mice were injected (i.p.) with the TcdB^FBD^ (20, 50, or 100 mg/kg) or TcdB^mu^ (20 mg/kg) twice a week for 5 weeks. Mice were injected intraperitoneally (i.p.) once with EdU at the dose of 100 mg/kg body weight 12 h before euthanization. Intestine tissues were cleaned with cold PBS, fixed in 4% formaldehyde, and embedded in paraffin. Four-μm-thick sections were prepared, and the intestine crypt proliferation was conducted using Click-iT EdU Alexa Fluor 594 Imaging Kit (C10339, Invitrogen) according to manufacturer’s introduction. Briefly, the sections were washed twice with 3% BSA in PBS and permeabilized in 0.5% Triton X-100, then incubated with a Click-iT reaction cocktail, followed by incubation in 5 μg/ml Hoechst 33342 according to the manufacturer’s protocol.

### Micro-computed tomography (μCT)

The right femur was extracted at euthanization and fixed in 4% paraformaldehyde for 2 days, then transferred into 70% ethanol. A 2.6-mm span of the distal femoral metaphysis was scanned on a desktop μCT (μCT-35; Scanco Medical AG) at 10-μm resolution using 50-kV peak tube potential and 151-ms integration time to measure cancellous 3D morphometric properties as previously described [62]. Standard trabecular bone parameters (BV/TV, Tb.N, Tb.Th) were calculated from each reconstructed stack through the metaphysis. Cortical thickness (Ct. Th) and area (Ct.Ar) were obtained from 20 slices reconstructed through the midshaft femur.

### Immunofluorescence staining

IF staining was performed on tissue sections that were fixed in 10% formalin (Fisher Scientific, Hampton, New Hampshire, USA) and embedded in paraffin. Antigen retrieval (Citrate buffer pH 6.0, 20 min boil in microwave oven) was performed before blocking and endogenous peroxidase activity was quenched on the slides intended for IF by incubation in 0.3% H_2_O_2_. Antibodies included: LEF1 (clone C12A5, #2230; Cell Signaling; 1:100), active β-catenin (clone D13A1, #8814; Cell Signaling; 1:100), K14 (PRB-155P, Covance, 1:400), K8 (MMS-162P, Covance, 1:200). For ERα and K5, IF staining was carried out by following standard procedures, after incubating in M.O.M blocking reagent (Vector Laboratories #BMK-2202) for 1 h at room temperature. Tissue sections were incubated with primary antibody for ERα (Santa Cruz #SC542, 1:100) and Cytokeratin 5 (K5) (BioLegend#905901, 1:1,000) diluted in M.O.M diluent reagent for 30 min at room temperature; the section was then washed with PBS and incubated with the secondary antibody [Goat anti-Rabbit IgG (H + L) Highly Cross-Adsorbed Secondary Antibody, Alexa Fluor 594 (Invitrogen A11037)] (for ERα primary antibody) and Goat anti-Chick IgG (H + L) Cross-Adsorbed Secondary Antibody, Alexa Fluor Plus 647 (Invitrogen A-32933)] (for Cytokeratin 5 primary antibody) for 30 min at room temperature. After washing with PBS again, the sections were stained with DAPI.

### Flow cytometry

Flow cytometric (FACS) analysis was performed after single cells were obtained using an Accuri C6 analyzer (BD Biosciences, San Jose, California, USA) and analyzed with CFlow software (BD Biosciences). The following antibodies were utilized: CD24 (clone M1/69, 564237; BD Biosciences; 1:100), CD29 (clone eBioHMb1-1, 12-0291-82; 1:250), FITC-TcdB^FBD^ (0.1 mg/ml), FZD7 (Clone 151143, FAB1981A; RD system; 1:100), CD31 (clone 390, 13-0311-85; eBioscience 1:100), CD45 (clone 30-F11, 13-0451-82; eBioscience; 1:100), and TER119 (clone Ter-119, 13-5921-85; eBioscience; 1:100). In some experiments, FACS analysis was performed using an LSR-II analyzer (BD Biosciences, San Jose, California, USA) and the data was further analyzed using Flowjo v10.8 Software (BD Life Sciences). First, the cells were incubated with Zombie Violet Fixable viability dye (1:200 dilution) for 20 min at room temperature in order to discriminate live and dead cells. Then, the dye dilute was washed out and the cells were proceeded with antibody staining. The following antibodies were used and purchased from Biolegend (San Diego, California, USA): anti-mouse CD31- BV605 (clone: 390, 102427, 1:50), anti-mouse CD45- BV605 (clone: 30-F11, 103140, 1:50), anti-mouse TER-119- BV605 (clone: TER-119, 116239, 1:50), anti-mouse CD24- PE (clone: 30-F1, 138503, 1:50), and anti-mouse CD29- PerCP-Cy5.5 (clone: HMβ1–1, 102227, 1:50). Original FCS files are publicly available: http://flowrepository.org/id/FR-FCM-Z6Q4.

### Tumorsphere formation and ex vivo tumorigenicity assays

Single cells obtained from cell lines or tumor tissues were resuspended and plated into round-bottom 96-well ultralow attachment plates (Corning) at a density of 1, 5, 10, 100, and 200 cells per well, or at 5,000 cells per well in 24-well flat-bottom ultralow attachment plates in the sphere culture medium (DMEM/F12 media supplemented with B27 (100 units/ml), insulin (10 μg/ml), EGF (20 ng/ml), and bFGF (20 ng/ml) with 150 nM TcdB^**FBD**^ or TcdB^**mu**^, PBS served as control. The frequency of TICs was calculated using the ELDA website (http://bioinf.wehi.edu.au/software/elda/index.html). The tumorsphere formation frequency in 24-well plates was calculated according to the formula F = Numbers of forming tumorspheres/Number of single cells plated. For secondary and tertiary tumorsphere formation, single cell suspensions prepared from the previous generation of tumorspheres were re-plated under the same conditions as the first generation.

### In vivo tumorigenicity assay

p53/BRCA1-deficient tumor organoid cells (10, 100, or 1,000 cells) were injected subcutaneously into nude mice in serum-free DMEM/Matrigel. The growth of tumor was evaluated daily over a 3-month period. The animal ethics endpoint was tumor reaching a size of 10 mm. Tumor volume was monitored and calculated as described above. The TIC frequency was derived as described above. For the Kaplan–Meier tumor-free survival curves, mice were considered tumor free until tumors were visible or palpable. For the Kaplan–Meier survival curves, mice were considered alive until tumors reach ethics endpoint a size of 10 mm.

### Reverse transcription and quantitative real-time PCR

Total RNAs from tumors were purified by either Trizol or the Allprep DNA/RNA mini kit (Qiagen). cDNA was generated with iScript (Bio-Rad, Berkeley, California, USA) according to the manufacturer’s protocol. Quantitative RT-PCR (qRT-PCR, for RNA) and PCR (for genomic DNA) were performed using FastStart SYBR Green Master (Roche, Indianapolis, Indiana, USA). PCR primers are listed below:

*FZD1*:forward:5′-GAGTTCTGGACCAGTAATCCGC-3′;reverse:5′-ATGAGCCCGTAAACCTTGGTG-3′; *FZD2*: forward: 5′-CTTCTCGCAAGAGGAGACTCG-3′; reverse: 5′-GTGGTGACCGTGAAGAAAGTG-3′; *FZD3*: forward: 5′- ATGGCTGTGAGCTGGATTGTC-3′; reverse: 5′-GGCACATCCTCAAGGTTATAGGT-3′; *FZD4*: forward: 5′-AACCTCGGCTACAACGTGAC-3′; reverse: 5′-GGCACATAAACCGAACAAAGGAA-3′; *FZD5*: forward: 5′-GGTGTGCCAGGAAATCACG-3′; reverse: 5′-CACAAGCGGCCAGAATTGG-3′; *FZD6*: forward: 5′-TCTGCCCCTCGTAAGAGGAC-3′; reverse: 5′-GGGAAGAACGTCATGTTGTAAGT-3′; *FZD7*: forward: 5′-GCCACACGAACCAAGAGGAC-3′; reverse: 5′-CGGGTGCGTACATAGAGCATAA-3′; *FZD8*: forward: 5′-GGGTTACCTGTTGGAAGTGAC-3′; reverse: 5′-GGCACCGTGATCTCTTGGC-3′; *FZD9*: forward: 5′-CGCACGCACTCTGTATGGAG-3′; reverse: 5′-GCCGAGACCAGAACACCTC-3′; *FZD10*: forward: 5′-CATGCCCAACCTGATGGGTC-3′; reverse: 5′-GCCACCTGAATTTGAACTGCTC-3′; *Gapdh*: forward: 5′-GGTGAAGGTCGGTGTGAACG-3′; reverse: 5′-CTCGCTCCTGGAAGATGGTG-3′; *Axin2*: forward: 5′-ATGAGTAGCGCCGTGTTAGTG-3′; reverse: 5′- GGGCATAGGTTTGGTGGACT -3′; *Vim*: forward: 5′-CGGCTGCGAGAGAAATTGC-3′; reverse: 5′-CCACTTTCCGTTCAAGGTCAAG-3′; *Zeb1*: forward: 5′-GCTGGCAAGACAACGTGAAAG-3′; reverse: 5′-GCCTCAGGATAAATGACGGC-3′; *Rnf43*: forward: 5′-CACGAGTTTCATCGAACGTGT-3′; reverse: 5′-CTGGCGAATGAGGTGGAGT-3′.

### Viability assay for human breast cancer organoids

Organoid lines derived from breast cancer patients are identified, as previously reported [50]. 74T was a luminal organoid line and 86T was a basal-like line. The organoid was cultured in 250 μl DMEM/F12 supplemented with 10 mM Hepes, 50 μg/ml Primocin, 1% GlutaMAX, 1:50 B27, 1 mM N-Acetylcysteine, 50 ng/ml EGF, 20 ng/ml FGF 2, 10 μM Y-27632, 100 ng/ml Noggin. TcdB^FBD^ or TcdB^mu^ (150, 300, 500, 1,000 nM) was added to the culture medium every day for 10 days, PBS was used as a vehicle control. The viability of organoids was assessed using the CellTiter-Glo luminescent cell viability assay (# G7570, Promega) according to the manufacturer’s protocol.

### Immunoblot

Tumor tissues were lysed by RIPA buffer (50 mM Tris, 1% NP40, 150 mM NaCl, 0.5% sodium deoxycholate, 0.1% SDS) with protease inhibitor cocktail (Roche). Total proteins were loaded on SDS-PAGE and transferred to PVDF membrane. After blocking, proteins were detected with a 1:1,000 dilution of primary antibody active β-catenin (clone D13A1, #8814; Cell Signaling; 1:1,000) using the enhanced chemiluminescence (ECL) method (Pierce).

### Statistical analysis

All statistical analyses were performed using GraphPad Prism 6.0 (San Diego, California, USA). An unpaired *t* test was used for comparisons between 2 experimental groups, and ANOVA was used for comparisons of more than 2 groups. Unless otherwise indicated, all results were averaged from biological triplicates and values are reported as means ± SEM. ***P*** < 0.05 was considered statistically significant.

## Supporting information

S1 Fig Expression levels of *FZDs* in human breast cancers. Expression levels of *FZDs* in different subtypes of human breast cancer based on bc-GenExMiner online tool.
(PDF)

S2 Fig Expression levels of *FZDs* in human breast cancer METABRIC cohort. Expression levels of *FZDs* in different subtypes of human breast cancers from the METABRIC cohort, based on cbioportal online tool. Subtypes: (A) Basal-like; (B) Claudin-low; (C) Her2; (D) LumA (Luminal A); (E) LumB (Luminal B); (F) NC (Not classified); (G) Normal-like.
(PDF)

S3 Fig Expression levels of *FZDs* in human breast cancer TCGA cohort. Expression levels of *FZDs* in different subtypes of human breast cancers from the TCGA, PanCancer Atlas cohort, based on cbioportal online tool. Subtypes: (A) Basal-like; (B) Her2; (C) Luminal A; (D) Luminal B; (E) Normal-like; (F) NA (not available).
(PDF)

S4 Fig Expression levels of *FZDs* in murine breast cancer models. High expression levels of *Fzd7* and *Fzd6* in mouse models for human basal-like/triple-negative breast cancer; data was based on GEO accession # GSE25488 (in heatmap, red to blue represents highest to lowest expression levels) [36]. The first 5 from the left side (marked as mammary tumor tissue rep1-5) represent the *C3(1)-Tag* model.
(PDF)

S5 Fig The percentage of FZD7^+^ cells are assessed in 3 breast cancer models. **(A)** Schematic diagram showing the generation of a p53/BRAC1-deficient breast cancer model by intraductal injection of *Ad-K8-Cre* into *Trp53*^*L/L*^*;Brca1*^*L/L*^*;R26Y* female mice. **(B)** Kaplan–Meier tumor-free results showing that *Trp53*^*L/L*^*;Brca1*^*L/L*^*;R26Y* female mice (*n* = 17) injected with *Ad-K8-Cre* developed malignant mammary tumors over time after *Ad-K8-Cre* injection. **(C)** Representative FACS plots of FZD7^+^ cells from normal mammary glands, *C3(1)-Tag*, p53/BRCA1-deficient, and *MMTV-PyMT* tumors. Numerical values are in S1 Data.
(PDF)

S6 Fig Co-immunofluorescence staining of select markers. Normal mammary gland (MG) and mammary tumors from the p53/BRCA1-deficient, *MMTV-PyMT*, and *C3(1)-Tag* models were stained with antibodies for K5 and ERɑ. Arrows indicate ER^+^ luminal mammary epithelial cells in the normal MG. Scale bar = 10 μm.
(PDF)

S7 Fig Full western blot. (PDF)

S8 Fig *FZD7* expression in normal human breast epithelial cells. Heatmap shown here is based on single-cell RNA sequencing data available at the Human Protein Atlas website (https://www.proteinatlas.org/). Luminal progenitors (LPs) are indicated as *SOX10*^+^*TP63*^-^ cells; hormone receptor (HR)+ luminal cells are indicated as *ESR1*^+^*FOXA1*^+^ cells; basal (B) cells are indicated as *TP63*^+^ cells.
(PDF)

S9 Fig TcdB^FBD^ inhibits Wnt signaling in human breast cancer cell lines. **(A)** Left panel: phylogenetic analysis of human FZD proteins [15]; right panel: qRT-PCR analysis of *Fzds* in MDA-MB-231 cells. **(B)** Cell viability measured by the MTT assay showed that TcdB^FBD^ did not exhibit cytotoxicity to the indicated human cell lines (error bars indicate mean ± SEM, 3 independent experiments). **(C)** Representative images of clonogenic growth of PaTu8988s and HPAF-II cells cultured in the presence of TcdB^FBD^ (150 nM) or LGK974 (100 nM). **(D)** Representative images of sphere formation assay in PaTu8988s and HPAF-II cells cultured in the presence of TcdB^FBD^ (150 nM) or LGK974 (100 nM). **(E)** Quantitation of colony numbers and sphere formation from (C and D), error bars indicate mean ± SEM, *n* = 4. **(F)** Immunoblot analysis of active β-catenin expression in PaTu8988s and HPAF-II cells cultured in the presence of TcdB^FBD^ (150 nM) or LGK974 (100 nM). Actin serves as a loading control. **(G)** Quantitative analysis of expression of active β-catenin expression in (F). NS, not significant;*, *p* < 0.05. Numerical values are in S1 Data.
(PDF)

S10 Fig CHIR99021 rescued TcdB^FBD^ inhibition on the growth of organoids derived from p53/BRCA1-deficient mouse mammary tumors. **(A)** Quantitation of organoids numbers in culture with or without CHIR99021(5 μM). **(B)** Quantitation of organoid sizes at day 5 in culture. Numerical values are in S1 Data.
(PDF)

S11 Fig TcdB^FBD^ inhibits Wnt signaling in p53/BRCA1-deficient mammary tumor cells. **(A)** Experiments were carried out as described in Fig 3A. qRT-PCR analysis showing down-regulation of Wnt signaling-related genes (*Axin2* and *Rnf43)* and EMT-associated genes (*Vim* and *Zeb1*) in tumor organoids upon TcdB^FBD^ treatment. **(B)** Experiments were carried out as described in Fig 3D. qRT-PCR analysis showing down-regulation of Wnt-associated genes (*Axin2* and *Rnf43*) and EMT-associated genes (*Vim* and *Zeb1*) in TcdB^FBD^-treated xenograft tumors. **(C)** Experiments were carried out as described in Fig 3D. Representative immunostaining images of LEF1 expression (green) in different treatment groups are shown (Control, TcdB^FBD^ or TcdB^mu^). Scale bar = 50 μm. Numerical values are in S1 Data.
(PDF)

S12 Fig TcdB^FBD^ inhibits growth of human basal-like breast cancer organoids. The viability of 2 lines of human breast cancer organoids, a luminal tumor line 74T (panel A) and a basal-like tumor line 86T (panel B), were exposed to the indicated concentrations of TcdB^FBD^ or TcdB^mu^ (0 nM, 150 nM, 300 nM, 500 nM, 1,000 nM). Cell viability was assessed using the CellTiter-Glo luminescent assay. Error bars were from 3 technical replicates. Numerical values are in S1 Data.
(PDF)

S13 Fig Tumor sphere/organoid-forming cells in p53/BRCA1-deficient mouse mammary tumors are largely FZD7^+^. **(A)** FACS analysis using FITC-TcdB^FBD^ and anti-FZD7 antibody showed that primary p53/BRCA1-deficient tumors cells targeted by TcdB^FBD^ are largely FZD7^+^. (**B**) Representative images showing the tumorspheres formed from p53/BRCA1-deficient tumor cells. **(C)** Tumorsphere cells cultured from p53/BRCA1-deficient tumors were examined by FACS analysis using an anti-FZD7 antibody. **(D)** Representative images showing the tumor organoid formed from p53/BRCA1-deficient tumor cells. **(E)** FACS analysis of FZD7^+^ cells in the tumor organoids described in (D) showing that most organoid cells express FZD7. Numerical values are in S1 Data.
(PDF)

S14 Fig TcdB^FBD^ and cisplatin showed synergistic effect in inhibiting basal-like mammary tumor organoids. **(A)** Representative images of p53/BRCA1-deficient mouse mammary tumor organoids treated with TcdB^FBD^ alone (150 nM), cisplatin alone (0.2 μm), or a combination of both. **(B)** Quantitation of organoids sizes for the indicated treatment groups in (A). **(C)** Quantitation of organoid numbers for the indicated groups in (A). **(D)** Representative pictures of *C3(1)-Tag* mouse mammary tumor organoids treated with TcdB^FBD^ alone, cisplatin alone, or a combination of both. **(E)** Quantitation of organoids sizes for the indicated treatment groups in (D). **(F)** Quantitation of organoid numbers for the indicated groups in (D). Scale bar = 100 μm. Numerical values are in S1 Data.
(PDF)

S1 Table Tumorsphere number following 3 passages of p53/BRCA1-deficient mammary tumor cells with 150 nM TcdB^FBD^ or TcdB^mu^ or PBS in the sphere culture medium. (DOCX)

S2 Table Analysis of the sphere-forming capabilities of p53/BRCA1-deficient mammary tumor cells using the limiting dilution assay. (DOCX)

S1 Data Excel sheet containing the data used in this manuscript to generate the graphs. (XLSX)

## Abbreviations

CRD: cysteine-rich domain
CSPG4: chondroitin sulfate proteoglycan 4
DMSO: dimethyl sulfoxide
ECL: enhanced chemiluminescence
EMT: epithelial–mesenchymal transition
ER: estrogen receptor
FACS: fluorescence-activated cell sorting
GSK3: glycogen synthase kinase-3
IACUC: Institutional Animal Care and Use Committee
IF: immunofluorescence
LEF1: lymphocyte enhancer-binding factor 1
LP: luminal progenitor
LRP1: LDL receptor–related protein 1
PAM: palmitoleic acid
PR: progesterone receptor
PVRL3: poliovirus receptor-related 3
qRT-PCR: quantitative real-time PCR
RBC: red blood cell
WT: wild type
