# Supplementary material for: Targeted inhibition of Wnt signaling with a Clostridioides difficile toxin B fragment suppresses breast cancer tumor growth
Source: PLoS Biol. 2023 Nov 9;21(11):e3002353. doi: 10.1371/journal.pbio.3002353 (PMC10635564; doi:10.1371/journal.pbio.3002353)

Supplementary Fig. S1

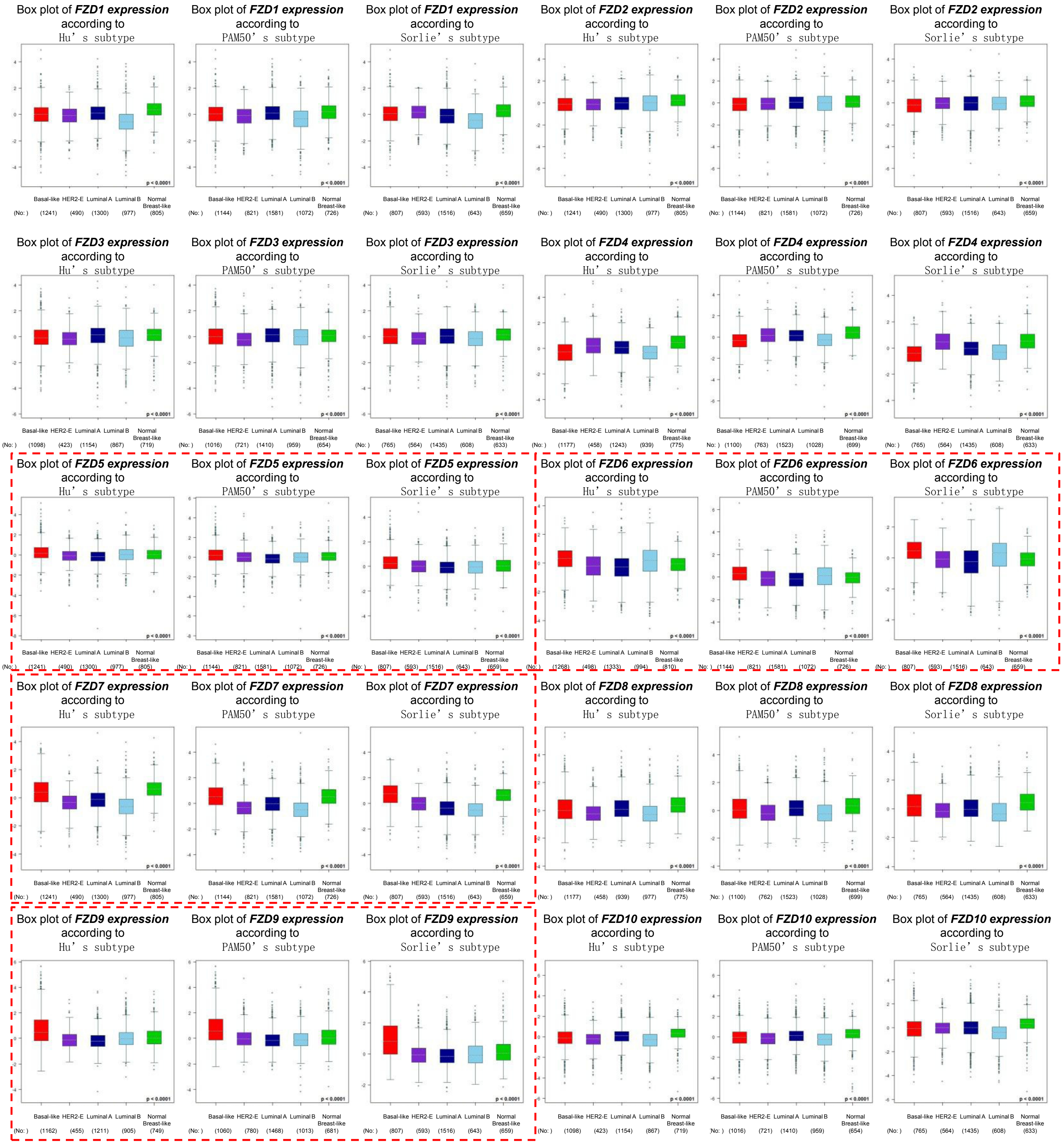

Supplement: S1 Fig — Expression levels of FZDs in different subtypes of human breast cancer based on bc-GenExMiner online tool. (PDF) [file pbio.3002353.s001.pdf]
