# Supplementary figures and images for: Targeted inhibition of Wnt signaling with a Clostridioides difficile toxin B fragment suppresses breast cancer tumor growth
Source: PLoS Biol. 2023 Nov 9;21(11):e3002353. doi: 10.1371/journal.pbio.3002353 (PMC10635564; doi:10.1371/journal.pbio.3002353)

Supplementary Fig. S2

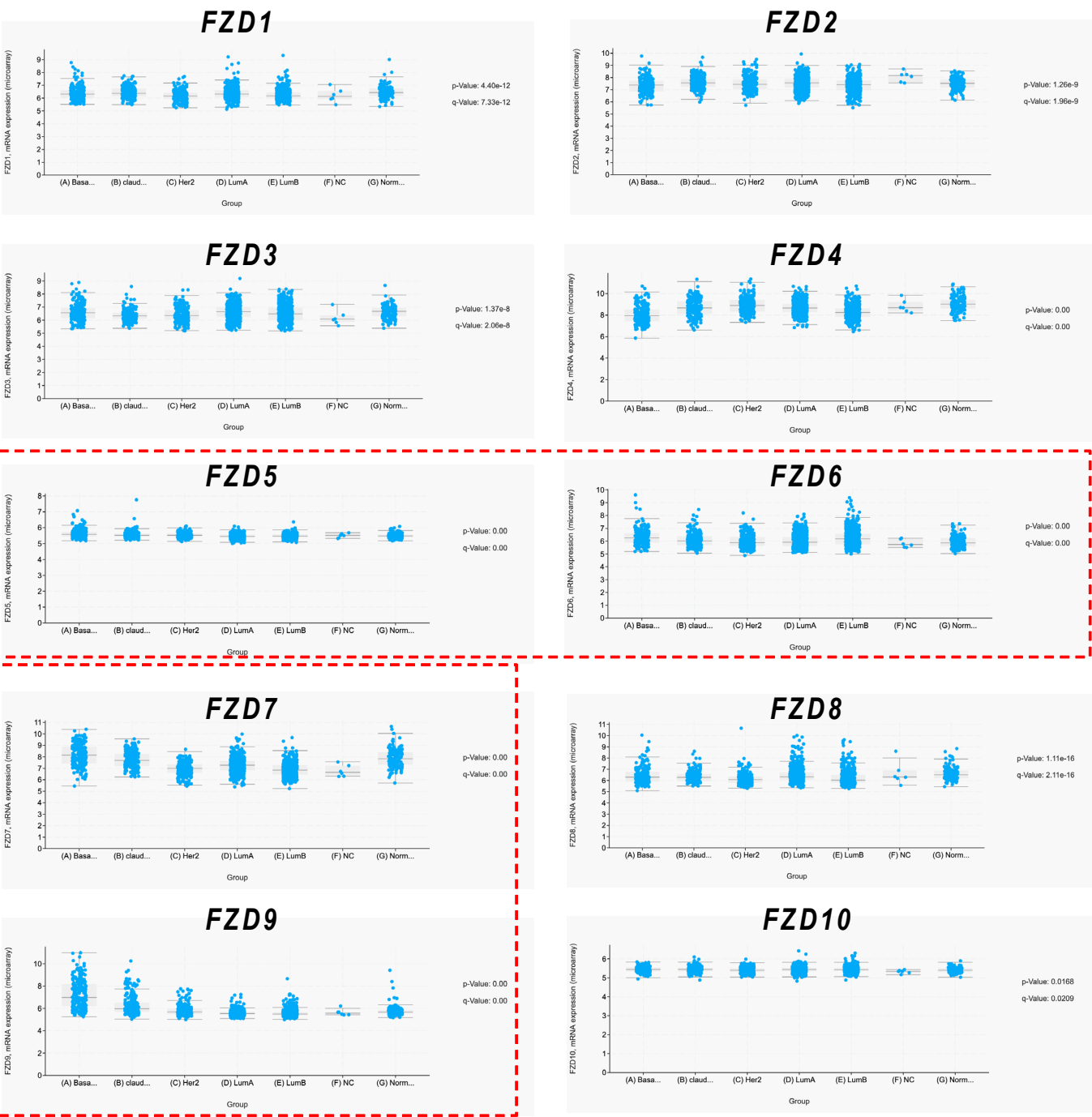

Supplement: S2 Fig — Expression levels of FZDs in different subtypes of human breast cancers from the METABRIC cohort, based on cbioportal online tool. Subtypes: (A) Basal-like; (B) Claudin-low; (C) Her2; (D) LumA (Luminal A); (E) LumB (Luminal B); (F) NC (Not classified); (G) Normal-like. (PDF) [file pbio.3002353.s002.pdf]

Supplementary Fig. S3

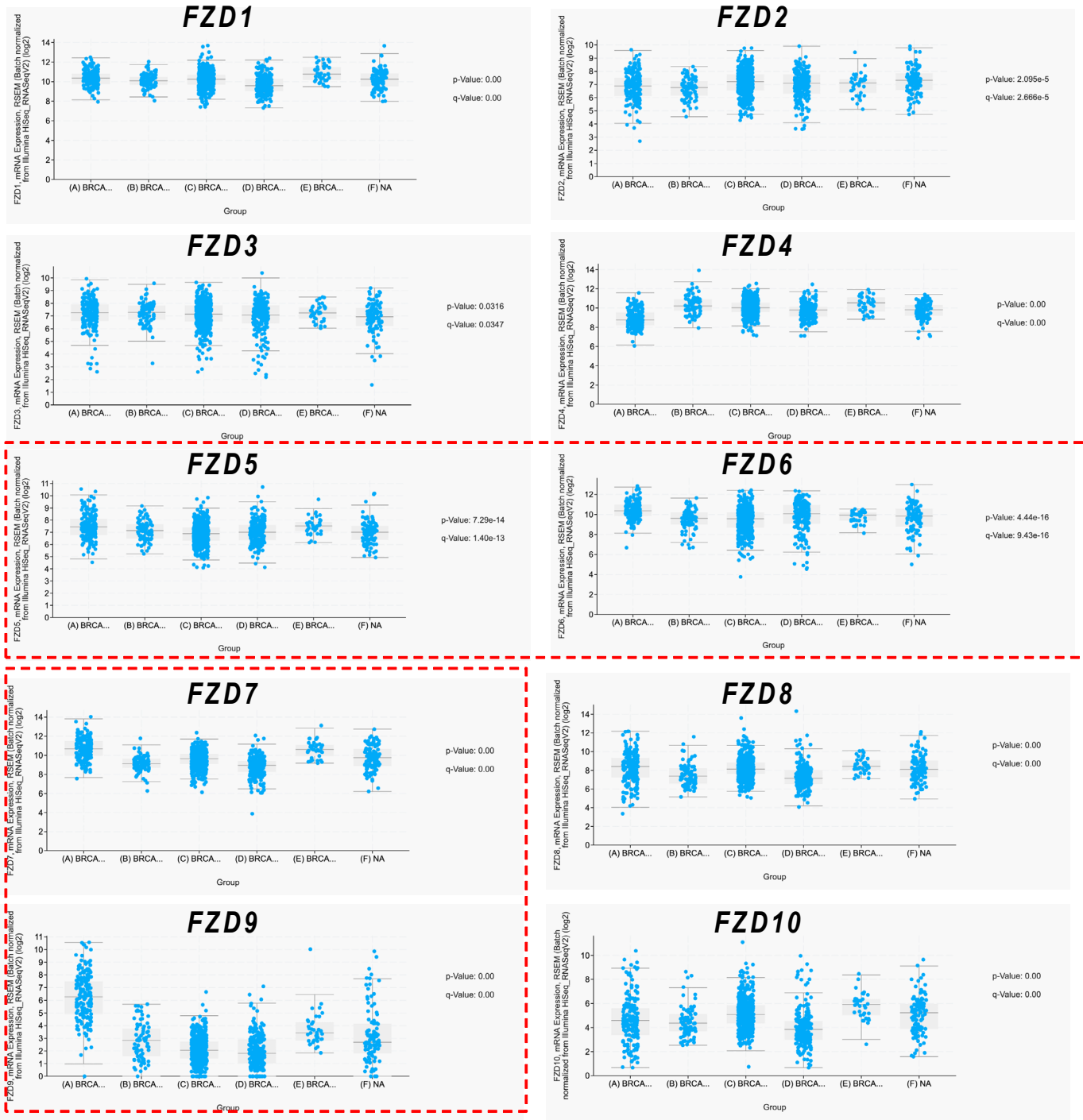

Supplement: S3 Fig — Expression levels of FZDs in different subtypes of human breast cancers from the TCGA, PanCancer Atlas cohort, based on cbioportal online tool. Subtypes: (A) Basal-like; (B) Her2; (C) Luminal A; (D) Luminal B; (E) Normal-like; (F) NA (not available). (PDF) [file pbio.3002353.s003.pdf]

Supplementary Fig. S4

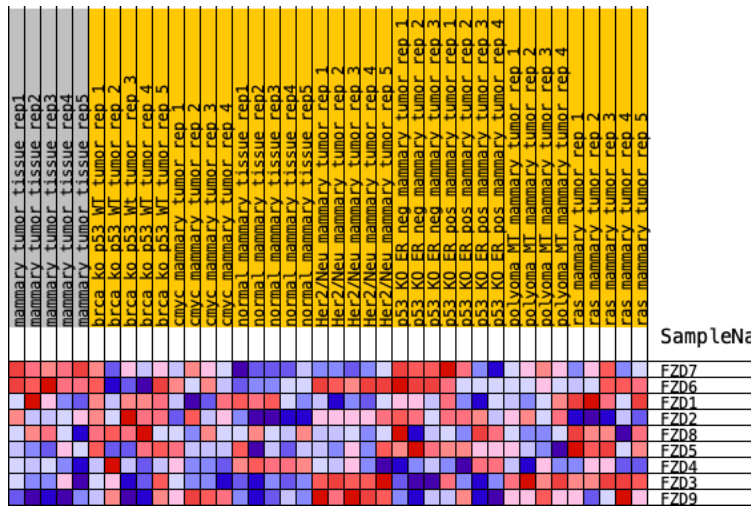

Supplement: S4 Fig — High expression levels of Fzd7 and Fzd6 in mouse models for human basal-like/triple-negative breast cancer; data was based on GEO accession # GSE25488 (in heatmap, red to blue represents highest to lowest expression levels) [36]. The first 5 from the left side (marked as mammary tumor tissue rep1-5) represent the C3(1)-Tag model. (PDF) [file pbio.3002353.s004.pdf]

## Supplementary Fig. S5

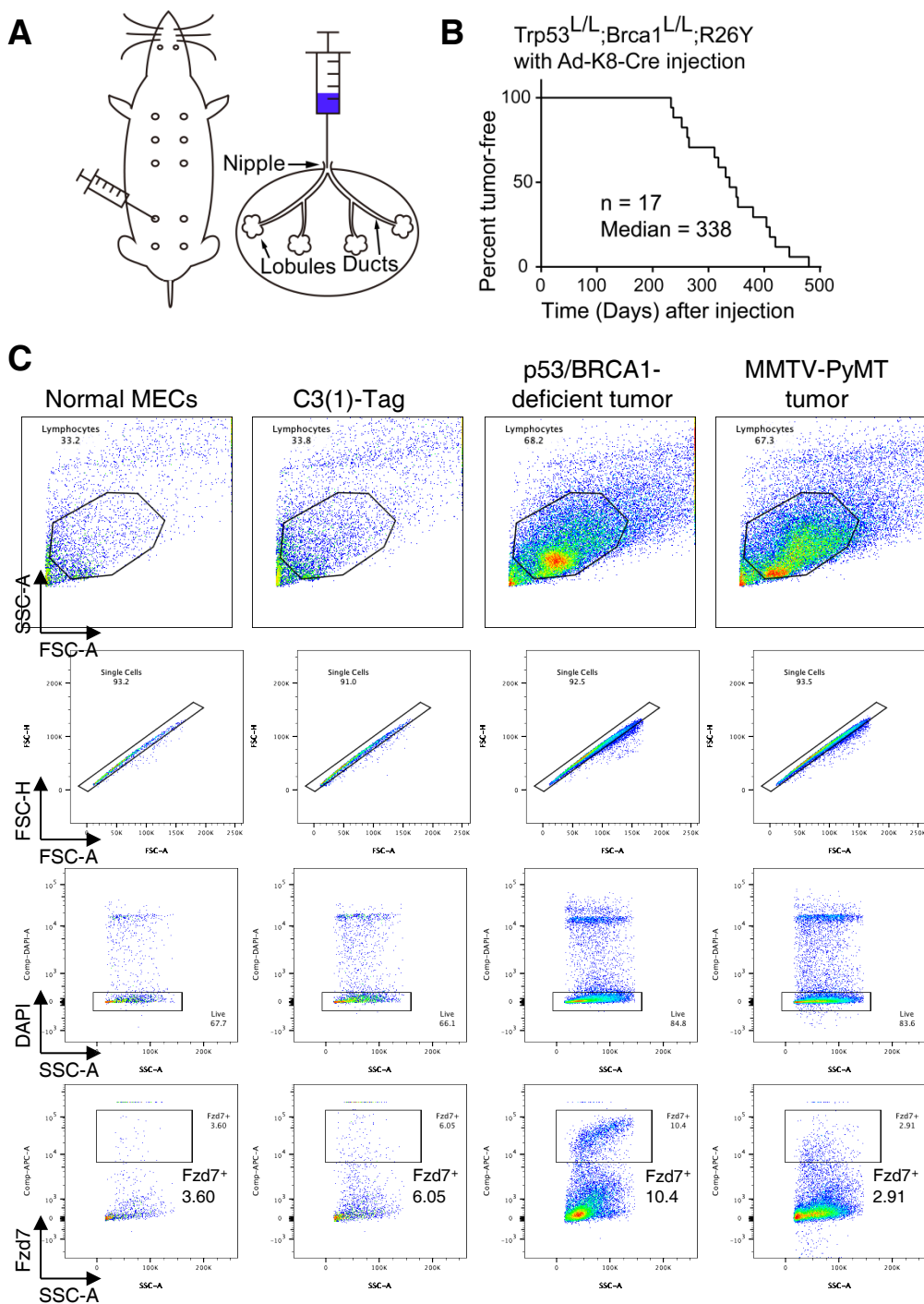

Supplement: S5 Fig — (A) Schematic diagram showing the generation of a p53/BRAC1-deficient breast cancer model by intraductal injection of Ad-K8-Cre into Trp53L/L;Brca1L/L;R26Y female mice. (B) Kaplan–Meier tumor-free results showing that Trp53L/L;Brca1L/L;R26Y female mice (n = 17) injected with Ad-K8-Cre developed malignant mammary tumors over time after Ad-K8-Cre injection. (C) Representative FACS plots of FZD7+ cells from normal mammary glands, C3(1)-Tag, p53/BRCA1-deficient, and MMTV-PyMT tumors. Numerical values are in S1 Data. (PDF) [file pbio.3002353.s005.pdf]

Supplementary Fig. S6

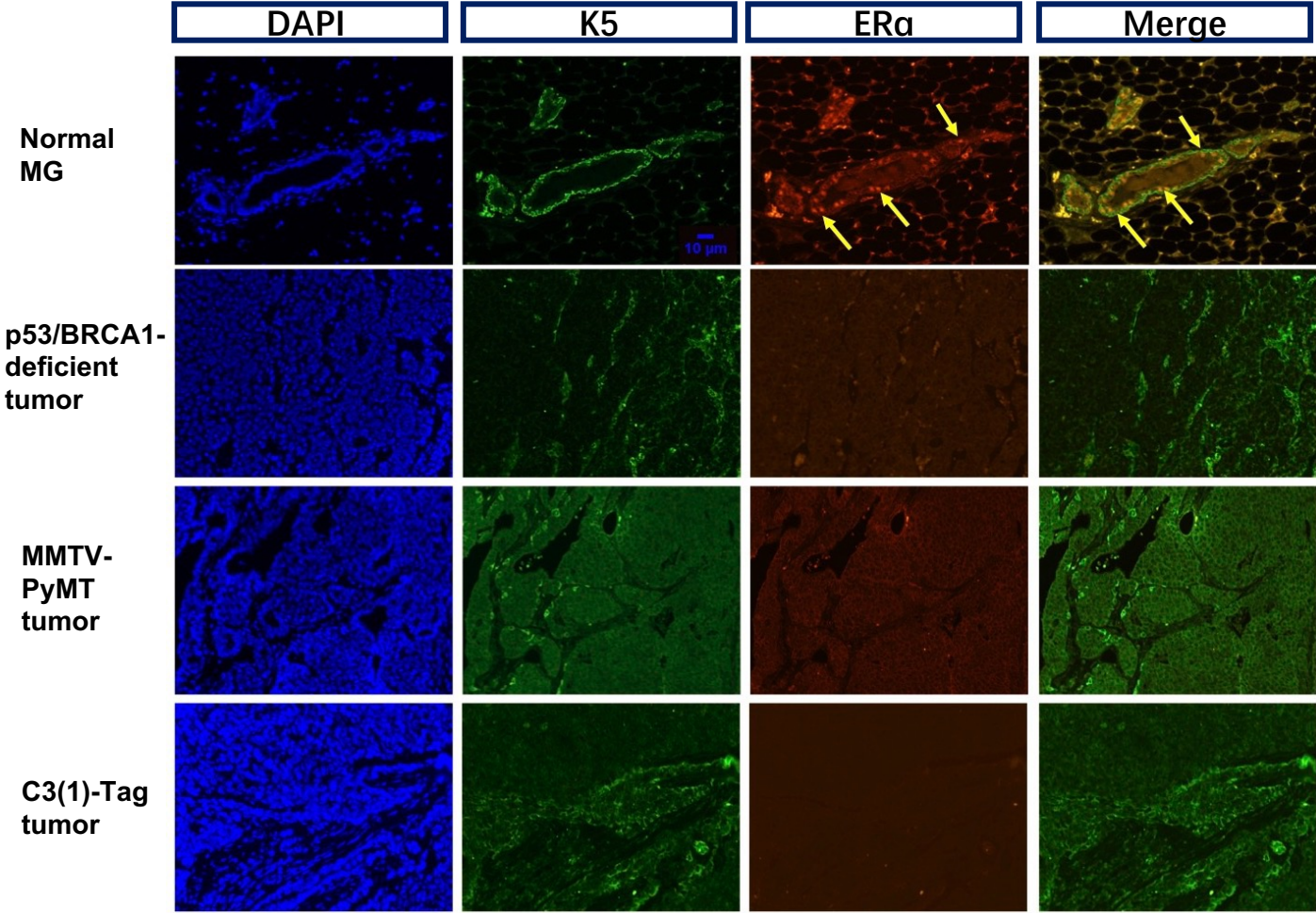

Supplement: S6 Fig — Normal mammary gland (MG) and mammary tumors from the p53/BRCA1-deficient, MMTV-PyMT, and C3(1)-Tag models were stained with antibodies for K5 and ERɑ. Arrows indicate ER+ luminal mammary epithelial cells in the normal MG. Scale bar = 10 μm. (PDF) [file pbio.3002353.s006.pdf]

### Supplementary Fig. S8

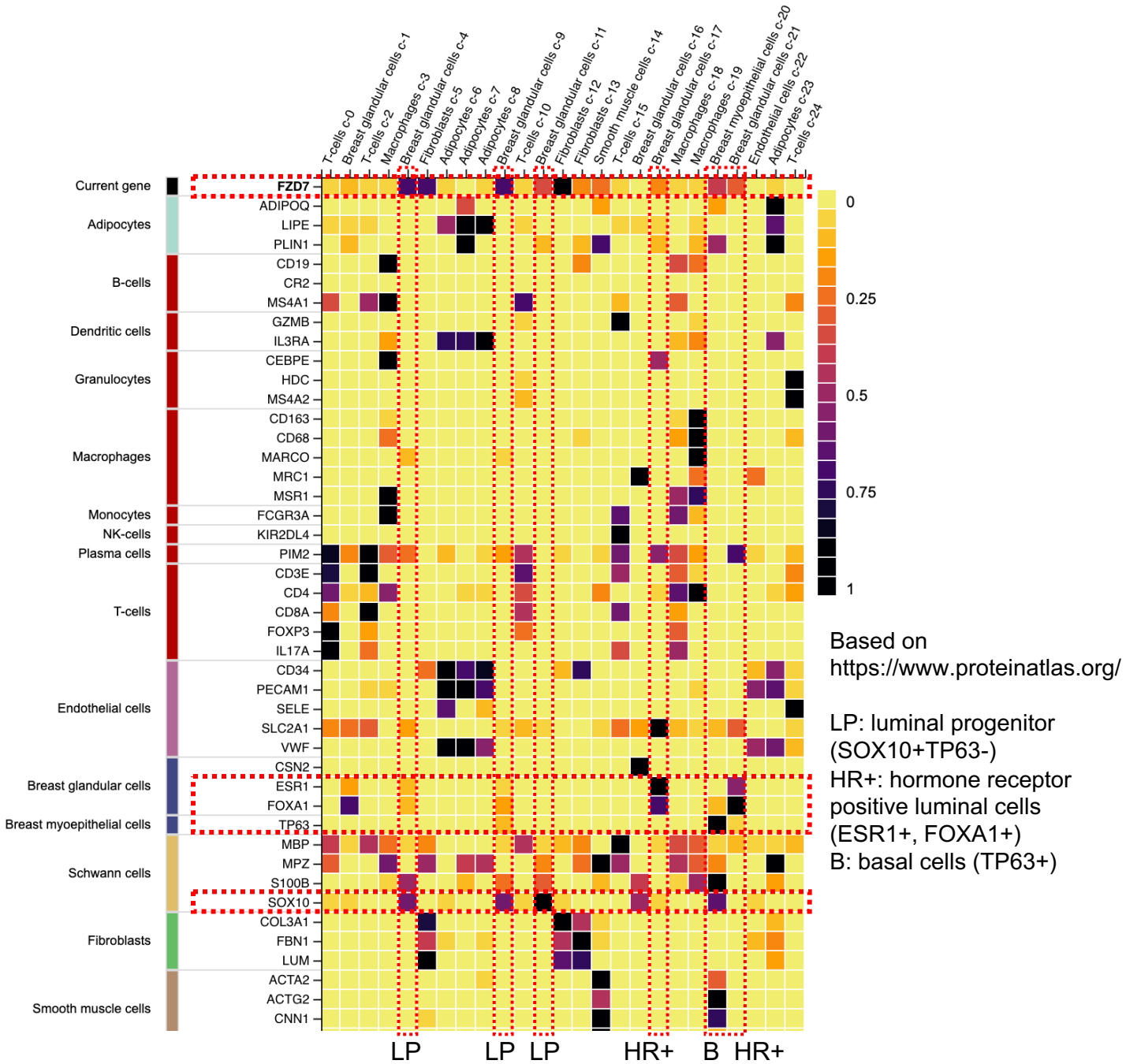

Supplement: S8 Fig — Heatmap shown here is based on single-cell RNA sequencing data available at the Human Protein Atlas website (https://www.proteinatlas.org/). Luminal progenitors (LPs) are indicated as SOX10+TP63- cells; hormone receptor (HR)+ luminal cells are indicated as ESR1+FOXA1+ cells; basal (B) cells are indicated as TP63+ cells. (PDF) [file pbio.3002353.s008.pdf]

Supplementary Fig. S9

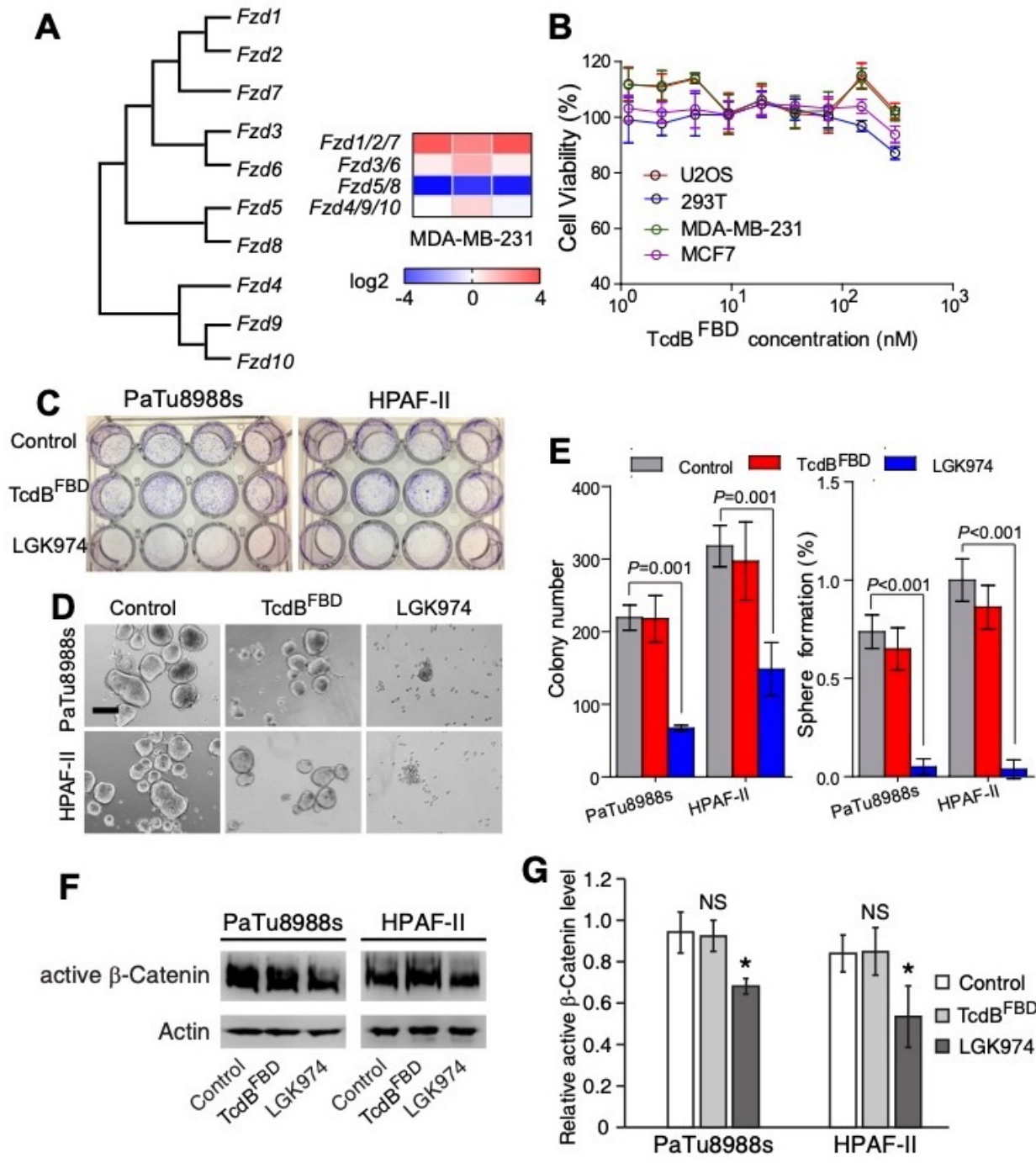

Supplement: S9 Fig — (A) Left panel: phylogenetic analysis of human FZD proteins [15]; right panel: qRT-PCR analysis of Fzds in MDA-MB-231 cells. (B) Cell viability measured by the MTT assay showed that TcdBFBD did not exhibit cytotoxicity to the indicated human cell lines (error bars indicate mean ± SEM, 3 independent experiments). (C) Representative images of clonogenic growth of PaTu8988s and HPAF-II cells cultured in the presence of TcdBFBD (150 nM) or LGK974 (100 nM). (D) Representative images of sphere formation assay in PaTu8988s and HPAF-II cells cultured in the presence of TcdBFBD (150 nM) or LGK974 (100 nM). (E) Quantitation of colony numbers and sphere formation from (C and D), error bars indicate mean ± SEM, n = 4. (F) Immunoblot analysis of active β-catenin expression in PaTu8988s and HPAF-II cells cultured in the presence of TcdBFBD (150 nM) or LGK974 (100 nM). Actin serves as a loading control. (G) Quantitative analysis of expression of active β-catenin expression in (F). NS, not significant;*, p < 0.05. Numerical values are in S1 Data. (PDF) [file pbio.3002353.s009.pdf]

Supplementary Fig. S10

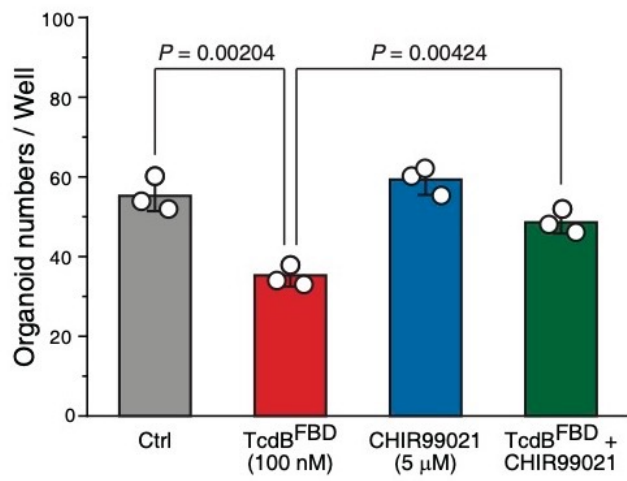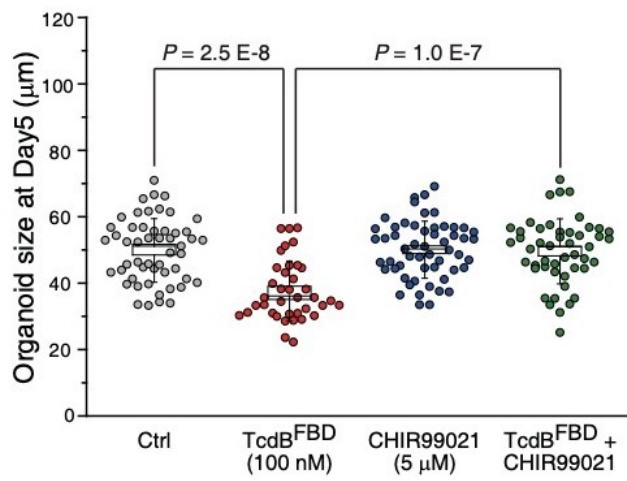

Supplement: S10 Fig — (A) Quantitation of organoids numbers in culture with or without CHIR99021(5 μM). (B) Quantitation of organoid sizes at day 5 in culture. Numerical values are in S1 Data. (PDF) [file pbio.3002353.s010.pdf]

Supplementary Fig. S11

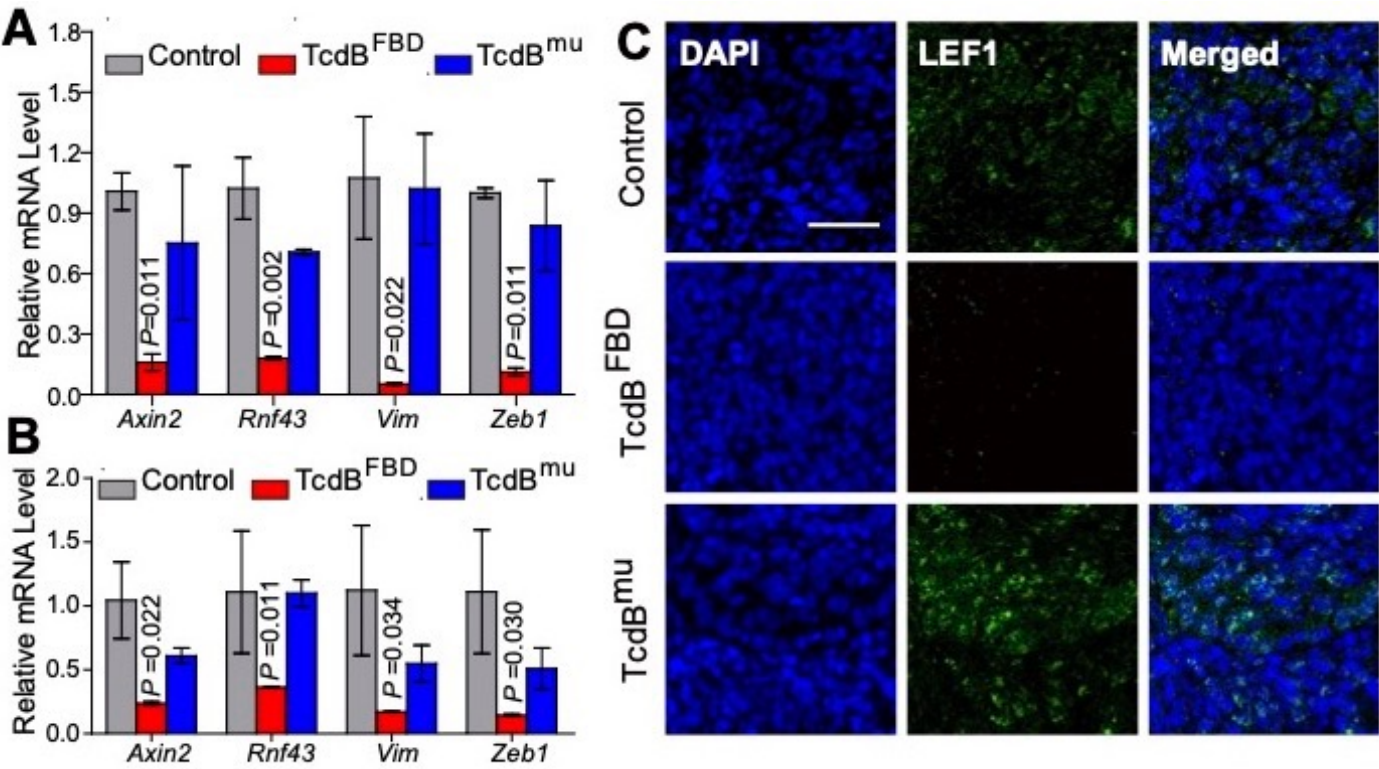

Supplement: S11 Fig — (A) Experiments were carried out as described in Fig 3A. qRT-PCR analysis showing down-regulation of Wnt signaling-related genes (Axin2 and Rnf43) and EMT-associated genes (Vim and Zeb1) in tumor organoids upon TcdBFBD treatment. (B) Experiments were carried out as described in Fig 3D. qRT-PCR analysis showing down-regulation of Wnt-associated genes (Axin2 and Rnf43) and EMT-associated genes (Vim and Zeb1) in TcdBFBD-treated xenograft tumors. (C) Experiments were carried out as described in Fig 3D. Representative immunostaining images of LEF1 expression (green) in different treatment groups are shown (Control, TcdBFBD or TcdBmu). Scale bar = 50 μm. Numerical values are in S1 Data. (PDF) [file pbio.3002353.s011.pdf]

Supplementary Fig. S12

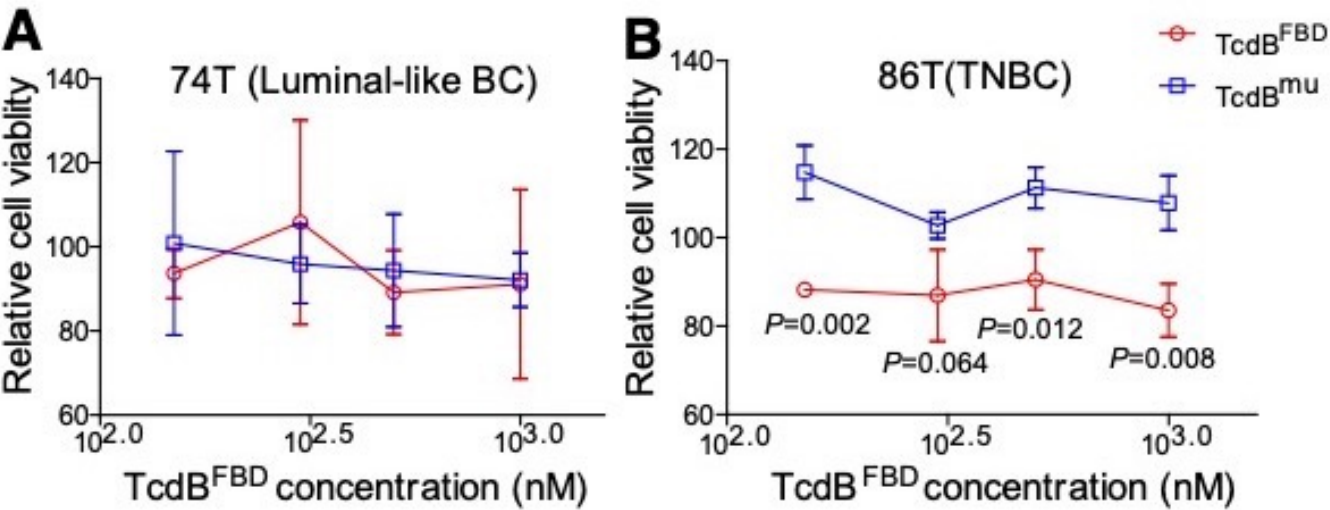

Supplement: S12 Fig — The viability of 2 lines of human breast cancer organoids, a luminal tumor line 74T (panel A) and a basal-like tumor line 86T (panel B), were exposed to the indicated concentrations of TcdBFBD or TcdBmu (0 nM, 150 nM, 300 nM, 500 nM, 1,000 nM). Cell viability was assessed using the CellTiter-Glo luminescent assay. Error bars were from 3 technical replicates. Numerical values are in S1 Data. (PDF) [file pbio.3002353.s012.pdf]

Supplementary Fig. S13

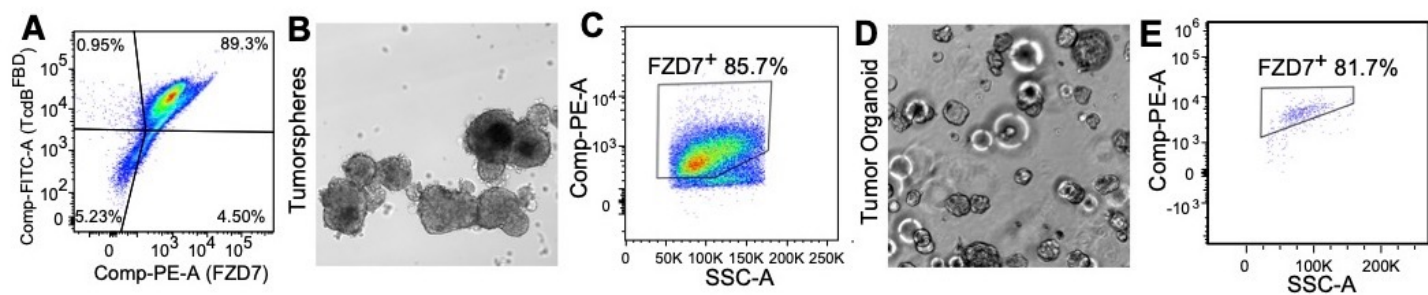

Supplement: S13 Fig — (A) FACS analysis using FITC-TcdBFBD and anti-FZD7 antibody showed that primary p53/BRCA1-deficient tumors cells targeted by TcdBFBD are largely FZD7+. (B) Representative images showing the tumorspheres formed from p53/BRCA1-deficient tumor cells. (C) Tumorsphere cells cultured from p53/BRCA1-deficient tumors were examined by FACS analysis using an anti-FZD7 antibody. (D) Representative images showing the tumor organoid formed from p53/BRCA1-deficient tumor cells. (E) FACS analysis of FZD7+ cells in the tumor organoids described in (D) showing that most organoid cells express FZD7. Numerical values are in S1 Data. (PDF) [file pbio.3002353.s013.pdf]

Supplementary Fig. S14

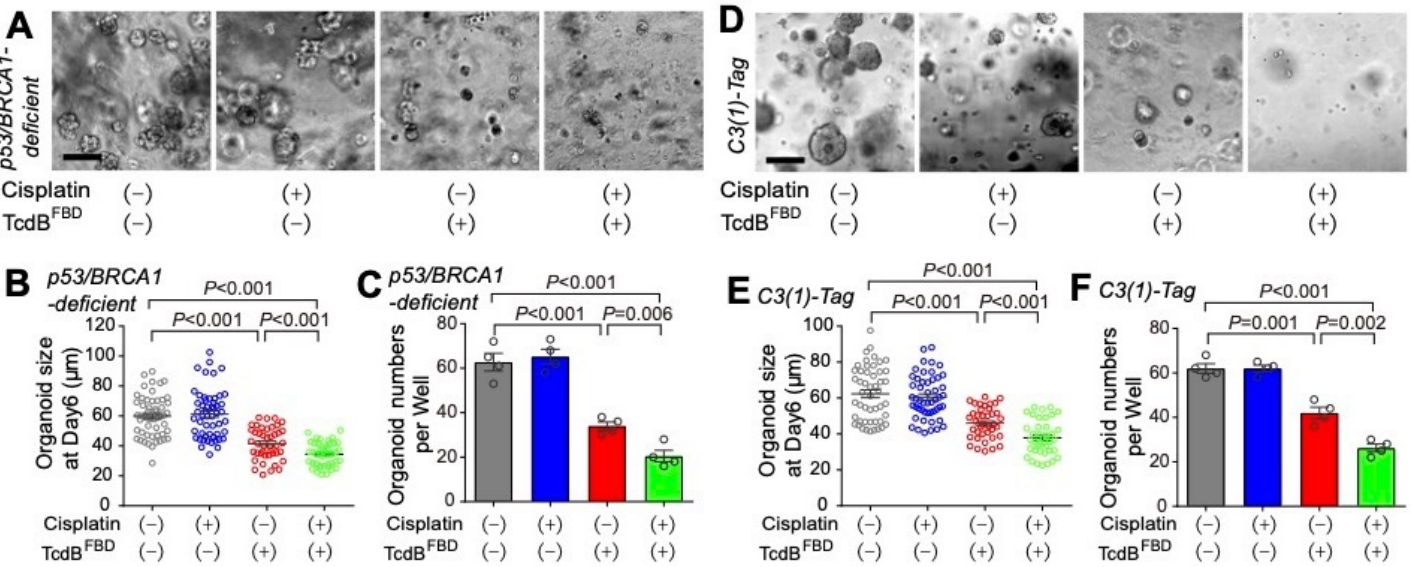

Supplement: S14 Fig — (A) Representative images of p53/BRCA1-deficient mouse mammary tumor organoids treated with TcdBFBD alone (150 nM), cisplatin alone (0.2 μm), or a combination of both. (B) Quantitation of organoids sizes for the indicated treatment groups in (A). (C) Quantitation of organoid numbers for the indicated groups in (A). (D) Representative pictures of C3(1)-Tag mouse mammary tumor organoids treated with TcdBFBD alone, cisplatin alone, or a combination of both. (E) Quantitation of organoids sizes for the indicated treatment groups in (D). (F) Quantitation of organoid numbers for the indicated groups in (D). Scale bar = 100 μm. Numerical values are in S1 Data. (PDF) [file pbio.3002353.s014.pdf]
