# Supplementary material for: Targeted inhibition of Wnt signaling with a Clostridioides difficile toxin B fragment suppresses breast cancer tumor growth
Source: PLoS Biol. 2023 Nov 9;21(11):e3002353. doi: 10.1371/journal.pbio.3002353 (PMC10635564; doi:10.1371/journal.pbio.3002353)

Supplementary Fig. S7

Full WB blots for Fig. 1C

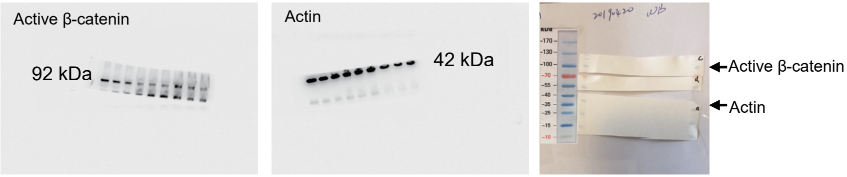

Full WB blots for Supplementary Fig. S9F

PaTu_ Active β-catenin (left 3); PaTu_Actin (left 3);

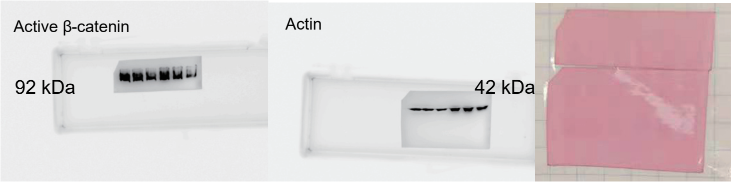

HPAF_ Active β-catenin (right 3); HPAF_Actin (right 3).

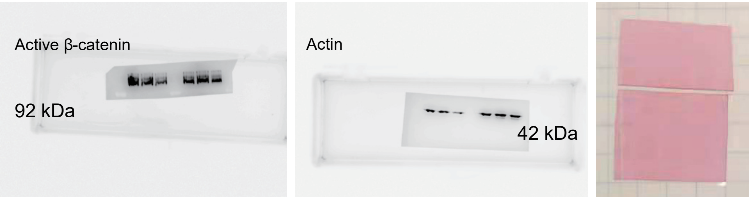

Supplement: S7 Fig — (PDF) [file pbio.3002353.s007.pdf]
