# Supplementary material for: Targeted inhibition of Wnt signaling with a Clostridioides difficile toxin B fragment suppresses breast cancer tumor growth
Source: PLoS Biol. 2023 Nov 9;21(11):e3002353. doi: 10.1371/journal.pbio.3002353 (PMC10635564; doi:10.1371/journal.pbio.3002353)
Supplement: S1 Table — (DOCX) [file pbio.3002353.s015.docx]

**Supplementary Table 1**. Tumorsphere number following three passages of p53/BRCA1-deficient mammary tumor cells with 150 nM TcdB^FBD^ or TcdB^mu^ or PBS in the sphere culture medium.

|  | Seeding cell  number | Control | TcdB^FBD^ | TcdB^mu^ |
| --- | --- | --- | --- | --- |
|  |  | Sphere number | | |
| Primary | 5000 | 22.00±2.61 | 11.38±5.95 | 19.00±4.00 |
| Secondary | 2000 | 14.69±2.93 | 0.73±1.14 | 5.40±3.34 |
| Tertiary | 2000 | 13.50±1.51 | 0.50±0.71 | 11.86±2.67 |
