# Supplementary material for: Targeted inhibition of Wnt signaling with a Clostridioides difficile toxin B fragment suppresses breast cancer tumor growth
Source: PLoS Biol. 2023 Nov 9;21(11):e3002353. doi: 10.1371/journal.pbio.3002353 (PMC10635564; doi:10.1371/journal.pbio.3002353)
Supplement: S2 Table — (DOCX) [file pbio.3002353.s016.docx]

**Supplementary Table 2**. Analysis of the sphere-forming capabilities of p53/BRCA1-deficient mammary tumor cells using the limiting dilution assay.

| Seeding cell  number | Control | TcdB^FBD^ | | CHIR99021 | TcdB^FBD^ +CHIR99021 |
| --- | --- | --- | --- | --- | --- |
|  |  | | Tumor sphere incidence | | |
| 2000 | 3/3 | 5/6 | | 3/3 | 6/6 |
| 1000 | 3/3 | 3/6 | | 3/3 | 5/6 |
| 500 | 5/6 | 1/6 | | 5/6 | 3/6 |
| 100 | 2/6 | 0/6 | | 3/6 | 1/6 |
| 10 | 0/6 | 0/6 | | 1/6 | 0/6 |
| Repopulating frequency | 1/266 | 1/1519 | | 1/194 | 1/576 |
| 95% CI | 1/(557-127) | 1/(2953-781) | | 1/(419-90) | 1/(1054-315) |
| *P (vs control)* | - | 0.0007 | | 0.548 | 0.121 |
| *P (vs TcdB^FBD^)* | - | - | | 0.00004 | 0.0351 |

Cells were seeded in the presence of 150 nM TcdB^FBD^ or CHIR99021 or TcdB^FBD^ plus CHIR99021 or PBS vehicle. Sphere formation was counted 15 days post-seeding. The frequency of sphere-forming cells (TICs) was calculated using the ELDA website (<http://bioinf.wehi.edu.au/software/elda/index.html>).
